# A certified plasmid reference material for the standardisation of *BCR–ABL1* mRNA quantification by real-time quantitative PCR

**DOI:** 10.1038/leu.2014.217

**Published:** 2014-08-12

**Authors:** H White, L Deprez, P Corbisier, V Hall, F Lin, S Mazoua, S Trapmann, A Aggerholm, H Andrikovics, S Akiki, G Barbany, N Boeckx, A Bench, M Catherwood, J-M Cayuela, S Chudleigh, T Clench, D Colomer, F Daraio, S Dulucq, J Farrugia, L Fletcher, L Foroni, R Ganderton, G Gerrard, E Gineikienė, S Hayette, H El Housni, B Izzo, M Jansson, P Johnels, T Jurcek, V Kairisto, A Kizilors, D-W Kim, T Lange, T Lion, K M Polakova, G Martinelli, S McCarron, P A Merle, B Milner, G Mitterbauer-Hohendanner, M Nagar, G Nickless, J Nomdedéu, D A Nymoen, E O Leibundgut, U Ozbek, T Pajič, H Pfeifer, C Preudhomme, K Raudsepp, G Romeo, T Sacha, R Talmaci, T Touloumenidou, V H J Van der Velden, P Waits, L Wang, E Wilkinson, G Wilson, D Wren, R Zadro, J Ziermann, K Zoi, M C Müller, A Hochhaus, H Schimmel, N C P Cross, H Emons

**Affiliations:** 1 National Genetics Reference Laboratory (Wessex), Salisbury District Hospital, Salisbury, UK; 2 Faculty of Medicine, University of Southampton, Southampton, UK; 3 European Commission, Joint Research Centre, Institute for Reference Materials and Measurements, Geel, Belgium; 4 Department of Haematology, Aarhus University Hospital, Aarhus, Denmark; 5 Hungarian National Blood Transfusion Service, Budapest, Hungary; 6 Regional Genetics Laboratory, Birmingham Women's NHS Foundation Trust, Birmingham, UK; 7 Department of Molecular Medicine and Surgery, Clinical Genetics Karolinska Institutet, Stockholm, Sweden; 8 Department of Laboratory Medicine, UZ Leuven, Belgium; 9 Department of Oncology, KU Leuven, Belgium; 10 Molecular Malignancy Laboratory and Haemato-Oncology Diagnostic Service, Cambridge University Hospitals NHS Foundation Trust, Cambridge, UK; 11 Haematology Department, Belfast City Hospital, Belfast, UK; 12 Haematology Laboratory and EA3518, University Hospital Saint-Louis, AP-HP, University Paris Diderot, Paris, France; 13 Department of Molecular Haematology, Yorkhill NHS Trust, Glasgow, UK; 14 Molecular Haematology, Bristol Royal Infirmary, Bristol, UK; 15 Hematopathology Unit, Hospital Clinic, IDIBAPS, Barcelona, Spain; 16 Department of Clinical and Biological Science, University of Turin, Turin, Italy; 17 Laboratoire Hematologie, CHU Bordeaux, Hematopoiese Leucemique et Cibles Therapeutiques, INSERM U1035, Universite Bordeaux, Bordeaux, France; 18 Combined Laboratories, Derriford Hospital, Plymouth, UK; 19 Department of Genetics and Molecular Pathology, SA Pathology, Adelaide, SA, Australia; 20 Imperial Molecular Pathology, Centre for Haematology, Imperial College London, London, UK; 21 Molecular Pathology, University Hospitals Southampton NHS Foundation Trust, Southampton, UK; 22 Hematology, Oncology and Transfusion Medicine Center, Vilnius University Hospital Santariskiu Clinics, Vilnius, Lithuania; 23 Laboratory of Molecular Biology and UMR5239, Centre Hospitalier Lyon-Sud, Hospices Civils de Lyon, Pierre Bénite, France; 24 Medical Genetics Department, Erasme Hospital, Brussels, Belgium; 25 Department of Clinical Medicine and Surgery, University ‘Federico II' of Naples, Naples, Italy; 26 Department of Immunology, Genetics and Pathology, Uppsala University, Uppsala, Sweden; 27 Department of Clinical Genetics, University and Regional Laboratories, Lund, Sweden; 28 Department of Internal Medicine-Hematology and Oncology, Masaryk University and University Hospital Brno, Brno, Czech Republic; 29 Turku University Hospital, TYKSLAB, Laboratory of Molecular Genetics, Turku, Finland; 30 Laboratory for Molecular Haemato-Oncology, Kings College Hospital, London, UK; 31 Cancer Research Institute, The Catholic University of Korea, Seoul, South Korea; 32 Abteilung für Hämatologie und internistische Onkologie, Universität Leipzig, Leipzig, Germany; 33 Children's Cancer Research Institute/LabDia Labordiagnostik and Medical University, Vienna, Austria; 34 Institute of Hematology and Blood Transfusion, Prague, Czech Republic; 35 Department of Experimental, Diagnostic and Specialty Medicine, University of Bologna, Bologna, Italy; 36 Cancer Molecular Diagnostics, St James's Hospital, Dublin, Ireland; 37 VU Medical Centre, Department of Haematology, Amsterdam, The Netherlands; 38 Department of Medical Genetics, NHS-Grampian, Aberdeen, UK; 39 Department of Laboratory Medicine, Medical University of Vienna, Vienna, Austria; 40 Laboratory of Hematology, Sheba Medical Center, Tel Hashomer, Israel; 41 Molecular Oncology Diagnostics Unit, Guy's Hospital, London, UK; 42 Lab Hematologia, Hospital de la Santa Creu i Sant Pau, Barcelona, Spain; 43 Division of Pathology, Rikshospital, Oslo University Hospital, Oslo, Norway; 44 Molecular Diagnostics Laboratory, Department of Hematology, University Hospital Bern, Bern, Switzerland; 45 Genetics Department, Institute of Experimental Medicine (DETAE), Istanbul University, Istanbul, Turkey; 46 Specialized Haematology Laboratory, Division of Internal Medicine, Department of Haematology, University Medical Centre, Ljubljana, Slovenia; 47 Department of Internal Medicine, Hematology/Oncology, Goethe University, Frankfurt, Germany; 48 Laboratoire d'hématologie, CHU Lille, Lille, France; 49 United Laboratories of Tartu University Hospitals, Tartu, Estonia; 50 Molecular Haematology Laboratory, PathWest Laboratory Medicine, Royal Perth Hospital, Perth, WA, Australia; 51 Hematology Department, Jagiellonian University, Krakow, Poland; 52 Hematology Department, Fundeni Clinical Institute, University of Medicine and Pharmacy ‘Carol Davila', Bucharest, Romania; 53 Hematology Department and HCT Unit, G. Papanicolaou Hospital, Thessaloniki, Greece; 54 Department of Immunology, Erasmus MC, Rotterdam, The Netherlands; 55 Bristol Genetics Laboratory, Southmead Hospital, Bristol, UK; 56 Department of Haematology, Royal Liverpool University Hospital, Liverpool, UK; 57 HMDS, Leeds Institute of Oncology, St James's University Hospital, Leeds, UK; 58 Sheffield Diagnostic Genetics Service, Sheffield Children's NHS Foundation Trust, Sheffield, UK; 59 Molecular Diagnostics, The Royal Marsden NHS Foundation Trust, Sutton, UK; 60 Department of Laboratory Diagnostics, Clinical Hospital Center, Zagreb University School of Medicine, Zagreb, Croatia; 61 Department of Hematology/Oncology, Jena University Hospital, Jena, Germany; 62 Haematology Research Laboratory, Biomedical Research Foundation, Academy of Athens, Athens, Greece; 63 III. Medizinische Klinik, Medizinische Fakultät Mannheim der Universität Heidelberg, Mannheim, Germany

## Abstract

Serial quantification of *BCR–ABL1* mRNA is an important therapeutic indicator in chronic myeloid leukaemia, but there is a substantial variation in results reported by different laboratories. To improve comparability, an internationally accepted plasmid certified reference material (CRM) was developed according to ISO Guide 34:2009. Fragments of *BCR–ABL1* (e14a2 mRNA fusion)*, BCR* and *GUSB* transcripts were amplified and cloned into pUC18 to yield plasmid pIRMM0099. Six different linearised plasmid solutions were produced with the following copy number concentrations, assigned by digital PCR, and expanded uncertainties: 1.08±0.13 × 10^6^, 1.08±0.11 × 10^5^, 1.03±0.10 × 10^4^, 1.02±0.09 × 10^3^, 1.04±0.10 × 10^2^ and 10.0±1.5 copies/μl. The certification of the material for the number of specific DNA fragments per plasmid, copy number concentration of the plasmid solutions and the assessment of inter-unit heterogeneity and stability were performed according to ISO Guide 35:2006. Two suitability studies performed by 63 *BCR–ABL1* testing laboratories demonstrated that this set of 6 plasmid CRMs can help to standardise a number of measured transcripts of e14a2 *BCR–ABL1* and three control genes (*ABL1, BCR* and *GUSB*). The set of six plasmid CRMs is distributed worldwide by the Institute for Reference Materials and Measurements (Belgium) and its authorised distributors (https://ec.europa.eu/jrc/en/reference-materials/catalogue/; CRM code ERM-AD623a-f).

## Introduction

The *BCR–ABL1* fusion gene is the primary pathogenic driver of chronic myeloid leukaemia (CML) and also characterizes a subset of patients with acute lymphoblastic leukaemia. As well as being of diagnostic importance, *BCR–ABL1* also serves as a specific marker of the malignant clone, and many laboratories worldwide routinely use serial reverse-transcription quantitative PCR (RT-qPCR) analysis to monitor the response of, individual CML or acute lymphoblastic leukaemia, patients to treatment.^1, 2, 3^ Indeed, international recommendations for the management of CML include key time-dependent therapeutic milestones based in part on such molecular monitoring.^4^

For routine testing, two measurements are typically made for each sample under investigation: an estimate of the number of *BCR–ABL1* transcripts as a measure of the burden of leukaemia and also the number of transcripts of an internal reference or control gene (CG) as a measure of overall quantity and quality of cDNA. Results for specimens that test positive for *BCR–ABL1* are expressed as the ratio of *BCR–ABL1*/CG transcript numbers in the same volume of cDNA, subject to previously described performance criteria.^5,6^ For samples that test negative for *BCR–ABL1*, the number of CG transcripts gives an indication of the sensitivity with which residual disease can be excluded for that particular specimen.^1^ However, despite the established clinical utility of RT-qPCR for monitoring of CML patients, the comparability of results between testing laboratories may vary widely.^7^ A major contributor to this variability is the use of different CGs to normalise results.

To help improve the comparability of results, an International Scale (IS) for *BCR–ABL1* was proposed,^8^ which is gradually being implemented by testing laboratories worldwide, most commonly by the derivation of laboratory-specific conversion factors (CFs) or the use of IS-calibrated kits or reagents.^9, 10, 11^ The IS expresses results as a percentage relative to the standardised baseline established in the International Randomized Study of Interferon and STI571 study; for example, major molecular response (MR), which corresponds to a 3-log reduction from the standardised baseline, is defined as 0.1% *BCR–ABL*^IS^.^8,12^ However, the IS was conceived at a time when most patients had RT-qPCR detectable disease and a major clinical consideration was whether a patient had or had not achieved major MR.^13^ Second-generation tyrosine kinase inhibitors result in faster and deeper responses compared with imatinib,^14,15^ and have prompted the need to define levels of deeper MR within the context of the IS. For example MR^4^, which corresponds to a 4-log reduction from the International Randomized Study of Interferon standardized baseline, has been defined as either (i) detectable disease ⩽0.01% *BCR–ABL*^IS^ or (ii) undetectable disease in cDNA with ⩾10 000 *ABL1* CG transcripts.^16^ Importantly, many patients treated with second-generation tyrosine kinase inhibitors (as well as an increasing proportion of patients treated long-term with imatinib)^17^ have undetectable *BCR–ABL1* mRNA by RT-qPCR and thus, it has become increasingly important for testing laboratories to estimate comparable and reliable numbers of CG transcripts. Indeed, recent data from the German CML-Study IV have shown that achievement of confirmed MR^4,5^ at 4 years predicted significantly higher survival probabilities compared with cases who only achieved 0.1–1% *BCR–ABL*^IS^.^17^

Determination of the number of *BCR–ABL1* and CG transcripts are typically performed by using a plasmid calibrator, however different calibrators (developed in house or commercially available) are in use worldwide and until now no common reference material exists to which they could be aligned. The aim of this study was to develop an internationally accepted plasmid certified reference material (CRM) that includes *BCR–ABL1* and the three most commonly used CGs (*ABL1*, *BCR* and *GUSB*) to help calibrate all measurements of residual disease in CML, and in particular, levels of deep MR.

## Materials and methods

### Preparation of individual certified plasmid solutions

**Figure 1:**
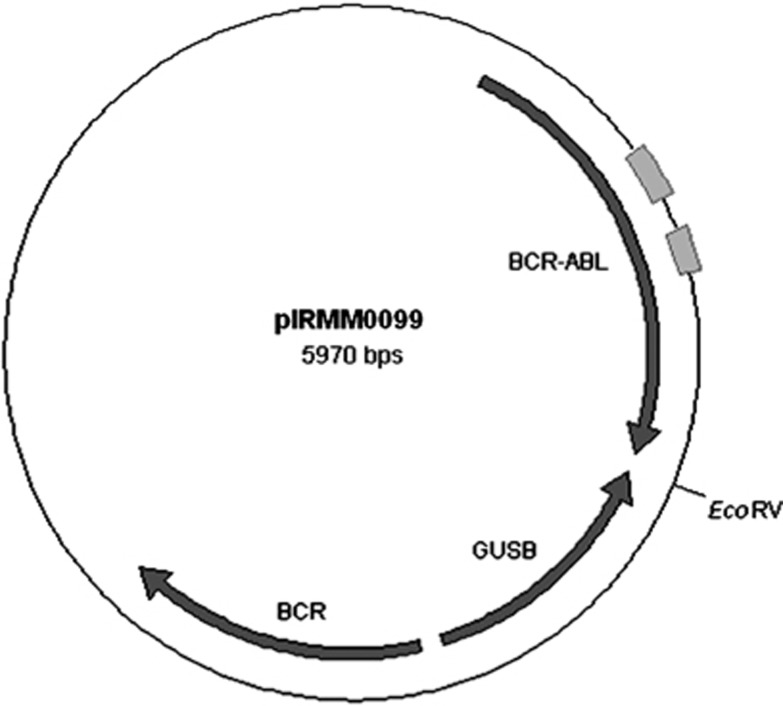
Schematic map of the multitarget plasmid pIRMM0099. The arrows represent the inserts from the transcript fragments of *BCR*, *GUSB* and *BCR–ABL1*. The rectangles show the location of the PCR targets *BCR–ABL1* and *ABL1* (the *ABL1* CG is within the *BCR–ABL1* fragment) used quantify the copy number concentration of the plasmid. The single restriction site for *Eco*RV is also shown.

Six plasmid solutions (ERM-AD623a-f), each with a different concentration level, were prepared starting from individual aliquots of the linearised stock pIRMM0099 plasmid (Figure 1 and Supplementary Methods). Dilutions were made in T_1_E_0.01_ buffer containing 50 ng/μl *Escherichia coli* tRNA (Sigma-Aldrich, Gillingham, UK) to yield a range spanning 10^6^^–10^ copies/μl. The plasmid solutions were sterilised by filtration with a 0.22-μm pore size hydrophilic polyethersulphone membrane (Merck Millipore, Watford, UK) and dispensed into high recovery polypropylene vials. A total of 5000 vials containing approximately 600 μl of plasmid solution were produced for each dilution.

### Digital PCR

Digital PCR was performed by three experienced laboratories: Institute for Reference Materials and Measurements, Geel, Belgium; LGC Limited, Molecular and Cell Biology Team, Teddington, UK and National Measurement Institute, Department of Innovation, Industry, Science and Research, Bioanalysis Group, West Lindfield, NSW, Australia. All three laboratories used the BioMark System (Fluidigm, South San Francisco, CA, USA) and the 12 756 digital array Integrated Fluidic Circuit, which comprises 765 individual partitions of approximately 6 nl volume each with total volume per panel of approximately 4.6 μl. Two PCR targets (one for *ABL1* and one for *BCR–ABL1* e14a2) were amplified in duplex reaction using the Europe Against Cancer primer/probe sets.^5,18^ Each sample was analysed on five panels of one digital array and the mean of these five results was considered as one measurement.

### Quantitative real-time PCR

Quantitative real-time PCR (qPCR) measurements to assess the homogeneity and stability were performed by the Institute for Reference Materials and Measurements, Geel, Belgium, using the ABI 7900 HT instrument (Applied Biosystems, Lennik, Belgium). The PCR conditions were the same as those used for the digital PCR measurements. For each concentration level, several vials were selected for the homogeneity and stability studies using a random stratified-sampling approach scheme for the whole batch. Each vial was measured three times in separate qPCR runs, and every measurement result was the mean result from three PCR wells (triplicate). A calibration curve with common plasmid solutions of pIRMM0099, produced independently from the stock solutions of ERM-AD623, was included in every qPCR run. This study design avoided 'between-run' effects by using a common calibrant for the calibration curves on each plate. The qPCR results of the *BCR–ABL1* PCR target showed the best method repeatability, so these results were used to assess the homogeneity and stability. The set-up of these experiments is described in detail in the certification report of ERM-AD623.^19^

### Statistical analysis

The certified copy number concentration of the six ERM-AD623 plasmid solutions was defined as the mean value of the accepted results from the digital PCR measurements. The combined expanded uncertainties associated with these copy number concentrations consist of uncertainties related to characterisation (*u*_char_), potential between-unit heterogeneity (*u*_bb_) and potential degradation during long-term storage(*u*_lts_). These different contributions were combined to estimate the expanded, relative uncertainty of the certified values (*u*_CRM, rel_) with coverage factor *k*:


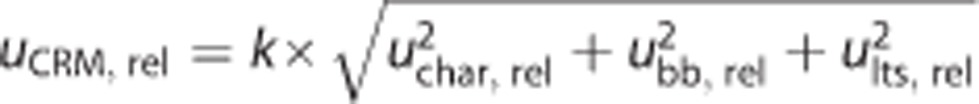


Based on the degrees of freedom of the different uncertainty contributions, a coverage factor *k* of 2 was applied to obtain the expanded uncertainties.^20^ The calculation of the individual uncertainty contributions (*u*_char_, *u*_bb_ and *u*_lts_) are described in the certification report.^19^

## Results

### Characterisation of copy number concentration

The certified values for the copy number concentration for the six plasmid solutions (ERM-AD623a-f) were determined by digital PCR measurements, carried out in three experienced laboratories. Eighteen vials of each concentration level were selected using a random stratified-sampling scheme. Each laboratory received six vials of each concentration level and was requested to provide six independent results, one per vial. The content of each vial was diluted gravimetrically and measured on different days and different arrays. For each concentration level, 18 independent results were obtained (Supplementary Table 4). Owing to the technical errors, two independent digital PCR results (one for ERM-AD623a and one for ERM-AD623f) were rejected. For each concentration level, the mean value of the accepted independent digital PCR results was assigned as the certified value for the copy number concentration of the plasmid (Table 1). The uncertainty related to this characterisation exercise, *u*_char_, was also calculated; no statistical difference between the results reported by the three laboratories was found (Supplementary Table 5).

### Assessment of homogeneity and stability

Homogeneity and stability studies for each concentration level were performed with qPCR measurements. Inter-unit homogeneity was evaluated to ensure that the certified copy number concentration of each plasmid solution was valid, within a stated uncertainty, for all vials produced for that concentration level. For each concentration level, 23 vials were analysed and statistical analysis of the results showed no outlying results or trends in the filling sequence (Supplementary Figure 3), suggesting that for each concentration level a homogeneous batch was produced. The relative uncertainty related to possible (undetected) heterogeneity, *u*_bb,rel_ was calculated for each concentration level (Table 1).

Stability testing was performed to establish conditions for long-term and short-term storage using an isochronous design.^21^ For the short-term stability study, 20 vials of each concentration level were selected and stored at 4 °C for 0, 1, 2 or 4 weeks. All six concentration levels were found to be stable at 4 °C for 4 weeks (Supplementary Figure 4). In the long-term stability study, the stability of each concentration level at −20 °C was tested for nine different time periods with a maximum of 24 months. Based on these results, it can be concluded that the plasmid CRMs can be stored at −20 °C (Supplementary Figure 5). The relative uncertainties related to the stability during long-term storage (24 months) at −20 °C, *u*_lts,rel_ were calculated for each concentration level and are listed in Table 1. In addition, the stability of the plasmid solutions after several freeze-thaw cycles was assessed in a small stability study, with a study design similar to the isochronous study design: 20 vials were exposed to 0, 5, 10 and 20  freeze-thaw cycles. The results obtained for the highest concentration level (ERM-AD623a) showed no significant degradation after 5 or 10  freeze-thaw cycles, however lower copy number concentrations were detected in the vials that were exposed to 20  freeze-thaw cycles. For the other concentration levels that were tested, no degradation was observed after 20  freeze-thaw cycles (Supplementary Figure 6). Although possible degradation was only observed for one concentration level, it was concluded that the plasmid solutions should not be exposed to more than 10  freeze-thaw cycles.

### Suitability

Two suitability studies were performed to test the performance of the plasmid solutions (as detailed in the Supplementary Methods). In the small scale study, nine laboratories were included which used 14 validated qPCR assays with one of the three CG: *ABL1*, *BCR* or *GUSB* (Supplementary Table 6). The results obtained with each of these 14 assays were considered as separate data sets. Laboratories were asked to generate 16 standard curves; 8 for their CG(s) of choice and 8 for *BCR–ABL1* e14a2, using two sets of ERM-AD623. In addition, each lab measured two common cDNA samples using the 16 calibration curves. The data were evaluated for their technical validity and some data were rejected due to one of the following reasons: deviations from the study protocol, degradation of cDNA samples during transport or too much variation^6^ within triplicate measurements, suggesting pipetting errors. For one data set, all results were rejected due to technical reasons. For the remaining 13 data sets, 174 individual calibration curves were accepted and evaluated for their slope and co-efficient of determination (*r*^2^). In total, 171/174 (98.3%) calibration curves had a slope within the range of −3.1 to −3.6; *BCR–ABL1* (*n*= 84), *ABL1* (*n*=36), *BCR* (*n*=23) and *GUSB* (*n*=28). Three (1.7%) calibration curves had a gradient less than −3.60 (−3.61 (*n*=2), −3.62 (*n*=1)). All calibration curves had an *r*^2^ above 0.993.

For the cDNA sample with the highest level of *BCR–ABL1*, 11 data sets provided technically acceptable results. For the other cDNA sample, one data set had to be removed as the cDNA sample was thawed during transportation, and thus 10 data sets were accepted. The estimated copy number concentration of *BCR–ABL1* were equivalent among the different data sets obtained by qPCR methods based on the Taqman technology (Supplementary Figure 7). However, for the qPCR method based on the LightCycler technology the measured copy number concentration of *BCR–ABL1* had the tendency to be 1.5–2.0 times lower. When comparing the *BCR–ABL1/CG* copy number ratio measured with Taqman-based or LightCycler-based methods the difference was smaller, as the measured copy number concentrations of CG were also lower with the LightCycler-based methods. (Supplementary Figure 8). However, the number of data sets per CG were too low to obtain conclusive results. Based on this suitability study, it was concluded that the six CRMs performed satisfactorily and can be used to calibrate different qPCR measurements, by determining the copy numbers of *BCR–ABL1* e14a2, *ABL1*, *BCR* and *GUSB*.

**Figure 2:**
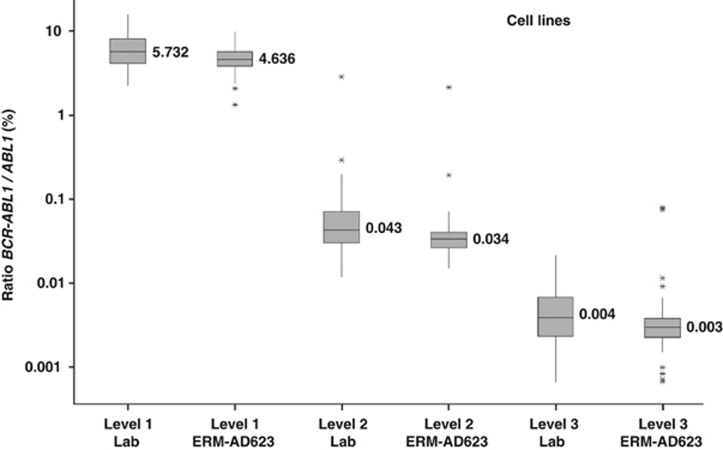
Comparison of measured % *BCR-ABL1/ABL1* ratio between laboratory calibrators (Lab) and ERM-AD623 for the cell line dilutions. Data from all centres in the second suitability study that used *ABL1* as a control gene are included; median values are indicated.

**Figure 3:**
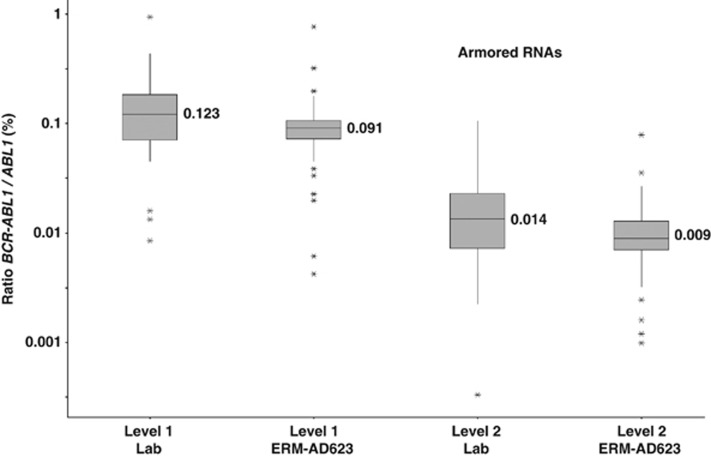
Comparison of measured % *BCR-ABL1/ABL1* ratio between laboratory calibrators (Lab) and ERM-AD623 for the aRNA mixtures. Data from all centres in the second suitability study that used *ABL1* as a control gene and returned aRNA data are included; median values are indicated.

A larger suitability study was performed where two different levels of *BCR–ABL1* e14a2 aRNA diluted in a background of *ABL1* aRNA predicted to correspond to approximately 0.1%, and 0.01% *BCR–ABL1/ABL1* plus three different HL60/K562 cell line lysates (~5%, 0.05%, 0.005% *BCR–ABL1*^IS^) were sent to 57 European laboratories. These laboratories used a variety of different assays (Supplementary Table 7) for analysis. Analysis of the cell line and aRNA samples indicated a good agreement between the *BCR–ABL1/ABL1* copy number ratios obtained with the laboratory calibrators and with ERM-AD623. Importantly, the degree of agreement between centres was significantly improved by the use of ERM-AD623 (Figures 2 and 3). As shown in Tables 2 and 3, the percentage of laboratories reporting results within twofold of the median or expected values was better when ERM-AD623 was used for all comparisons. Results within fivefold were equal or better when ERM-AD623 was used. It was not possible to perform a similar comparison for other CGs, as none of the participating laboratories used *BCR* and only six used *GUSB*.

**Figure 4:**
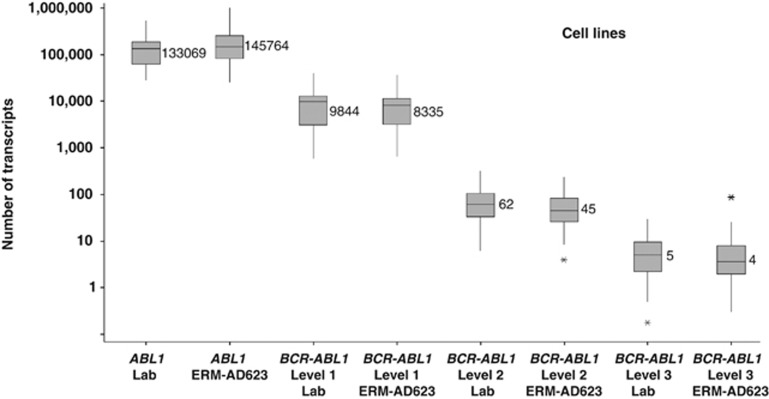
Reported numbers of *BCR–ABL1* and *ABL1* transcripts using laboratory-specific methods (which vary with regard to the amount of material analysed), laboratory calibrators (Lab) and ERM-AD623 for cell line dilutions. Data from all centres in the second suitability study that used *ABL1* as a control gene are included; median values are indicated. Values for *ABL1* are for all three dilutions combined; values for *BCR–ABL1* differ between the three levels and are shown separately.

**Figure 5:**
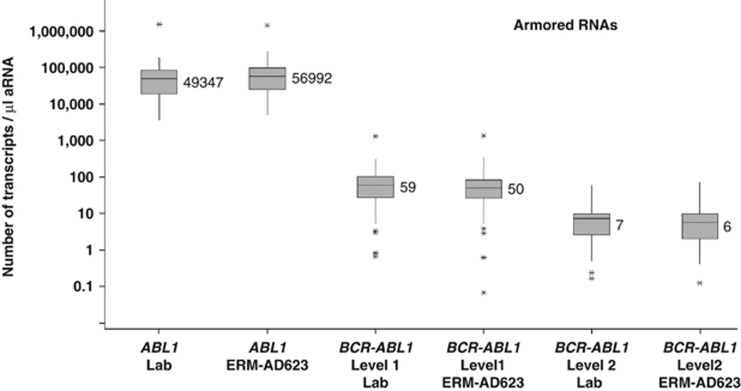
Estimates of copy numbers of *BCR–ABL1* and *ABL1* transcripts using laboratory calibrators and ERM-AD623 for the aRNA dilutions in the second suitability study. Individual laboratory protocols have been taken into account to derive estimate per μl of aRNA. Data from all centres in the second suitability study that used *ABL1* as a control gene and returned aRNA data are included; median values are indicated. Values for *ABL1* are for both dilutions combined; values for *BCR–ABL1* differ between the two levels and are shown separately. Expected values for *ABL1* if the lysis, reverse transcription and qPCR were all perfect is 305 500; expected *BCR–ABL1* values for levels 1 and 2 are 300 and 30, respectively.

Estimates of *BCR–ABL1* and *ABL1* copy number concentrations were very similar with laboratory calibrators and ERM-AD623 for both the cell line and aRNA dilutions (Figures 4 and 5). However, the aRNA samples were each predicted to contain 305 500 *ABL1* copies/μl, plus variable amounts of *BCR–ABL1*. When laboratory data were corrected for the amount of aRNA added to the PCR (which varied between centres as they were asked to use their routine protocols), the median number of *ABL1* copies per μl aRNA was 49 347 using the laboratory standard curve and 56 992 using ERM-AD623 as a standard curve.

## Discussion

Over the years, several different in-house and commercial plasmid calibrators for *BCR–ABL1* and CG measurement by RT-qPCR have been developed. Typically, these calibrators have independently assigned copy number concentrations based on their molecular weight and DNA concentration, but in the absence of an internationally accepted CRM it is inevitable that variation between calibrators has become established. Although the magnitude of this variation is not known, it is likely to adversely affect patient results since our study, as well as a previous study, both found that the use of a common plasmid calibrator substantially improves the comparability of test sample results between centres.^22^ Although it is likely that this variation may have been at least partly captured by laboratory-specific CFs, for samples with detectable disease, there is an increasing clinical need for laboratories to make comparable estimates of test sensitivity when *BCR–ABL1* is undetectable. Thus, there is an increasing need for laboratories to be able to make comparable and reliable estimates of CG transcript numbers.

We have developed a plasmid CRM, ERM-AD623, as a tool to help standardise the measurement of residual disease in CML. ERM-AD623 consists of a set of six plasmid solutions that are certified for the number of specific DNA fragments per plasmid and the copy number concentration of the plasmid. The number of specific DNA fragments per plasmid is defined by the sequence identity of the plasmid, as determined by dideoxy termination sequencing of the entire plasmid. The plasmid contains three inserts, which are all present as a single copy: one DNA fragment specific for the *BCR–ABL1* e14a2 transcript, one DNA fragment specific for the *GUSB* transcript and one DNA fragment specific for the *BCR* transcript (Supplementary Table 2). The insert from the *BCR–ABL1* e14a2 transcript also contains a large fragment from the native *ABL1* transcript. As a consequence, the copy number ratios *BCR–ABL1/ABL1*, *BCR–ABL1/GUSB* and *BCR–ABL1/BCR* of the plasmid are 1/1. The uncertainties related to these copy number ratios are considered to be negligible. The copy number concentration of the plasmid in the six plasmid solutions was determined by digital PCR. The expanded uncertainties, *u*_CRM_, associated with the certified copy number concentrations include the uncertainties related to characterisation, *u*_char_; potential between-unit heterogeneity, *u*_bb_; and potential degradation during long-term storage,*u*_lts_ (Table 1). Homogeneity was demonstrated and the conditions for storage were established by stability testing.

The suitability of ERM-AD623 as a calibrator for qPCR-methods quantifying the level of *BCR–ABL1* e14a2 transcript in cDNA samples was also investigated in two multicentre studies. The plasmid CRM is intended to calibrate the qPCR measurement and not the whole RT-qPCR process, including RNA extraction and reverse transcription. Therefore, no formal commutability studies could be performed. Instead, the suitability studies showed that the analytical behaviour (defined by the *r*^2^ and the slope of the calibration curve) of the plasmid solutions in different qPCR assays is within previously defined recommendations.^6,23^ In the small-scale suitability study, ERM-AD623 was used to calibrate qPCR measurements of two common cDNA samples. The *BCR–ABL1*/CG copy number ratios thus obtained were equivalent between the different qPCR assays used. However, when comparing the copy number concentration of *BCR–ABL1*, these results seem to confirm the previously reported variability between methods using TaqMan platforms versus methods using LightCycler platforms.^22^ This variation may be partly due to the lower input of cDNA in many LightCycler protocols and is likely captured by CFs for detectable disease for which a *BCR–ABL*1/CG ratio can be calculated. This difference might, however, may need to be taken into consideration in attempts to standardise measurement of undetectable disease. In the large-scale suitability study, we found that the use of ERM-AD623 improved the degree of agreement of results when *BCR–ABL1* is detectable for both cell lines and aRNAs. However, there were still large differences in estimates of CG copy numbers for the aRNA samples, for which the initial number of RNA molecules was known. Overall, the median estimates of *ABL1* and *BCR–ABL1* aRNA numbers were five- to sixfold lower than expected (Figure 5), suggesting that there is substantial room for most laboratories to improve the efficiency of reverse transcription and consequently the sensitivity of their assays.

ERM-AD623 is provided as a set of six plasmid solutions that should be used to construct calibration curves for both *BCR–ABL1* e14a2 and the CG of choice. As the sequences of *BCR–ABL1* and the CG are located on the same plasmid, the contribution of the calibrator to the measurement uncertainty associated with the measured value for the copy number ratio *BCR–ABL*1/*CG* is negligible. Furthermore, we anticipate that having *BCR–ABL1* and the CG on the same construct will help to reduce variability compared to assays that use different plasmid calibrators for different targets. Nevertheless, the uncertainty associated with the certified copy number concentrations of the ERM-AD623 solutions must be taken into account when reporting results.

Roughly 98% of CML patients express a p210 BCR*–*ABL protein, which is encoded in about half of cases by an e14a2 mRNA fusion (*BCR* exon 14 spliced to *ABL1* exon 2; also known as b3a2). A similar proportion expresses the smaller e13a2 (also known as b2a2) variant. About 10% of cases express both e14a2 and e13a2. The remaining 2% of cases are accounted for by multiple-atypical variants, the most common of which are e14a3, e13a3, e6a2, e19a2 and e1a2. Most (70%) cases of *BCR–ABL1-*positive acute lymphoblastic leukaemia cases express e1a2, with the remaining 30% split, expressing e14a2 and/or e13a2. As e14a2 and e13a2 only differ by 75bp, many laboratories use a common probe/primer set to amplify cDNA derived from both transcripts. However, it should be noted that ERM-AD623 is only certified for the measurement of e14a2 *BCR–ABL1*; for e13a2 the suitability of ERM-AD623 has to be verified at each laboratory by determining that e14a2 and e13a2 are amplified with equal efficiencies, which can be determined from the standard curve equations by looking specifically at the gradient and intercept components. Importantly, the certified copy number concentrations of ERM-AD623 refer to numbers of double-stranded plasmid molecules and thus should be doubled for use as a qPCR calibrator for single-stranded cDNA.

It is important to note that the use of ERM-AD623 does not by itself produce results on the IS; instead it helps to improve the accuracy of results prior to conversion as well as the accuracy of CG copy-number estimates for samples where *BCR–ABL1* is not detected. Conversion to the IS requires established CFs processes^9^ or the use of secondary reference materials that are traceable to the World Health Organisation International Genetic Reference Panel for the Quantification of *BCR–ABL* translocation.^10^ The combination of CFs and a common plasmid calibrator should help testing laboratories to generate standardized results. However, some lack of agreement between results from different laboratories using diverse methodologies and CGs will remain. Whether this remaining disagreement is acceptable, depends on the effect it has on clinical interpretation. When evaluating the performance characteristics of a method, two factors should be considered: trueness (that is the degree of closeness of mean measured-quantity value and the true-quantity value) and the precision (that is the degree to which repeated measurements under unchanged conditions show the same results). The trueness of a method can be estimated by comparing the average value obtained from several replicate measurements on a reference material with an established IS value. The precision of a method can be estimated from the 95% limit of agreement of all the individual measurement results obtained for the reference material.^24^ Existing experience with the set-up and validation of laboratory-specific CFs has shown that an average difference within ±1.2-fold of the established value and 95% limits of agreement within ±5-fold of the established value were achieved by the best performing methods.^9^ This led to an major MR concordance rate of 91%, a level of agreement which probably represents the maximum that can be achieved using the current RT-qPCR technology.

Although ERM-AD623 can be used by testing laboratories on a day-to-day basis, it may be used to align local plasmid calibrators, if that is preferred. It can also be used for assay optimisation, for example the cycle threshold or crossing point values for *BCR–ABL1* e14a2, and each CG should be identical for each dilution as they are present in a 1/1 ratio. It should be noted that the certified values refer to the use of 5 μl of each dilution; users may wish to use smaller volumes but the uncertainty of the certified value in that case would need to be determined and would be expected to be greater than that stated. In particular, for the lowest level dilution (CRM code ERM-AD623f) Poisson distribution effects would have to be taken into consideration. The plasmid dilutions can be obtained from the Institute for Reference Materials and Measurements or its authorised distributors (https://ec.europa.eu/jrc/en/reference-materials/catalogue/; CRM code ERM-AD623a-f)

## Figures and Tables

**Table 1:** Certified copy number concentrations of double-stranded plasmid DNA and their uncertainties for the six ERM-AD623 dilutions

*CRM*	*Copy number^a^ concentration of the plasmid (cp/μl)*	u*_char,rel_ (%)*	u*_bb,__rel_ (%)*	u*_lts__,__rel_ (%)*	u*_CRM__,rel_ (%)*	u*_CRM_ (cp/μl)*
ERM-AD623a	1.08 × 10^6^	3.28	3.57	2.75	11.15	0.13 × 10^6^
ERM-AD623b	1.08 × 10^5^	3.58	2.88	2.21	10.19	0.11 × 10^5^
ERM-AD623c	1.03 × 10^4^	3.01	2.59	2.81	9.73	0.10 × 10^4^
ERM-AD623d	1.02 × 10^3^	2.66	2.47	2.11	8.40	0.09 × 10^3^
ERM-AD623e	1.04 × 10^2^	3.06	2.75	2.43	9.56	0.10 × 10^2^
ERM-AD623f	10.0	4.28	4.37	3.83	14.42	1.5

Abbreviations: CRM, certified reference material; *u*_bb,rel_, relative uncertainity related to potential between-unit heterogeneity of the material; *u*_char,rel_, relative uncertainity related to the charecterisation study; *u*_CRM_, expanded uncertainity of the certified value (with *k*=2); *u*_CRM,rel_, relative expanded uncertainity of the certified value (with *k*=2); *u*_lts,rel_, relative uncertainity related to potential degradation during long-term storage (24 months at −20 °C).

a As the copy number concentrations refer to the copy numbers of double-stranded plasmid, these numbers should be doubled when calibrating quantitative real-time PCR experiments that measure single-stranded cDNA samples.

**Table 2:** Comparison of results for the cell line dilutions using local calibrators (Lab) and ERM-AD623

*Cell line dilution*	*Standard curve*	*Median value (% BCR–ABL1/CG)*	*% Labs within twofold*	*% Labs within fivefold*
Level 1	Lab	5.732	80.8	100
Level 2	Lab	0.043	75.0	96.1
Level 3	Lab	0.004	67.3	86.5
Level 1	ERM-AD623	4.636	92.3	100
Level 2	ERM-AD623	0.034	86.5	96.1
Level 3	ERM-AD623	0.003	80.8	94.2

**Table 3:** Comparison of results for the aRNA mixtures using local calibrators (Lab) and ERM-AD623

*aRNA mixture*	*Standard curve*	*Predicted value (% BCR–ABL1/CG)*	*% Labs within twofold*	*% Labs within fivefold*
Level 1	Lab	0.1	72	92
Level 2	Lab	0.01	64	94
Level 1	ERM-AD623	0.1	80	94
Level 2	ERM-AD623	0.01	72	92
