# Supplementary material for: A certified plasmid reference material for the standardisation of BCR–ABL1 mRNA quantification by real-time quantitative PCR
Source: Leukemia. 2014 Aug 12;29(2):369–76. doi: 10.1038/leu.2014.217 (PMC4320294; doi:10.1038/leu.2014.217)
Supplement: Supplementary Information [file leu2014217x1.doc]

**A CERTIFIED PLASMID REFERENCE MATERIAL FOR THE STANDARDISATION OF BCR-ABL1 mRNA QUANTIFICATION BY REAL TIME QUANTITATIVE PCR**

White, Deprez et al.,

**SUPPLEMENTARY METHODS:**

**Construction of multiple target plasmid pIRMM0099**

The multiple target plasmid was assembled in three consecutive DNA fragment insertions into the plasmid vector pUC18. DNA fragments specific for *BCR-ABL1* e14a2 (1397bp), *BCR* (963bp) and *GUSB* (813bp) transcripts were amplified from cDNA obtained from K562 cells (Hammersmith Hospital, London, UK) using primers shown in Supplementary Table 1. The fragments were designed to include commonly used *BCR-ABL1* and CG RT-qPCR amplicons. The *BCR* and *GUSB* PCR products were ligated into pCR2.1 (Invitrogen, Paisley, UK) to create pCR2.1_BCR and pCR2.1_GUSB and the multiple target plasmid was assembled into pUC18 using three consecutive DNA fragment insertions. pCR2.1_BCR was digested with *Eco*RI and the *BCR* insert was cloned into the *Eco*RI site of pUC18 (pUC18_BCR). pCR2.1_GUSB and pUC18_BCR were digested with *Xba*I and *Kpn*I and the *GUSB* insert was cloned into the *Xba*I/*Kpn*I sites of pUC18_BCR to create pUC18_BCR_GUSB. The 1397bp *BCR-ABL1* e14a2 product was digested with *Sal*I and cloned into pUC18_BCR_GUSB digested with *Sal*I to create pIRMM0099 (Figure 1).

**Preparation of stock solutions of pIRMM0099**

Stock solutions of pIRMM0099 were prepared using the QIAfilter Plasmid Maxi Kit (Qiagen, Manchester, UK). The DNA sequence of pIRMM0099 was confirmed by full sequencing in two laboratories and restriction enzyme digestion with *Hin*cII, *Pvu*II and *Xho*I (Supplementary Figures 1 and 2). Digital PCR studies also verified that only one copy of each insert was present in pIRMM0099 (Supplementary Table 2). pIRMM0099 was linearised using *Eco*RV and the DNA mass concentration of the linearised plasmid solution was determined using fluorometry (Picogreen dsDNA quantification kit, Molecular Probes Inc, Eugene, OR, USA), Based on DNA mass concentration (c_DNA_) the copy number of the plasmid solution was estimated by applying the following equation:


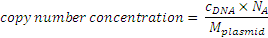


The calculation of the molar mass of pIRMM0099 (M_plasmid_) was based on the DNA sequence of the plasmid and the molar mass of each nucleotide. N_A_ is Avogradro’s constant. Two other DNA quantification techniques: spectrophotometry (ND-1000, NanoDrop Technologies, Wilmington, NC, USA) and digital PCR (see below for the description of the method) were used to confirm the DNA mass concentration measured by fluorometry. The estimated copy number concentrations of the plasmid in the stock solution using the three DNA quantification techniques were equivalent (Supplementary Table 3) excluding the presence of major contaminations of genomic DNA from the host bacterial cells in the plasmid solution. In addition, spectrophotometry was also used to assess the DNA purity by measuring the UV absorbance at 230 nm (A_230_), 260 nm (A_260_) and 280 nm (A_280_). The A_260_/A_280_ and A_260_/A_230_ ratios were 1.86 and 1.97 indicating that there were no major contaminations of proteins or aromatic substance from the nucleic acid extraction which might affect the behaviour of the plasmid in PCR reactions. The stock solution was diluted to 35 ng/µL (estimated copy number of 5.71 x 10^9^ copies/µL) in T_1_E_0.01_ buffer (1 mM Tris pH 8.0, 0.01 mM EDTA). The stock was stored in 8 aliquots at -20°C.

**Suitability studies**

Two multicenter studies were performed to test the performance of the plasmid CRMs as a calibrator for different qPCR assays measuring the level of *BCR-ABL1* e14a2 transcript in relation to the CG transcript in cDNA samples. Since the certified copy number concentrations of ERM-AD623 refer to copies of double stranded plasmid molecules, these values were doubled to quantify the *BCR-ABL1* and the CG transcript levels in cDNA samples which consist of single stranded DNA. Nine laboratories participated in the first small scale study using one or more validated qPCR method(s) to determine the level of *BCR-ABL1* e14a2 transcript in relation to one of the CG: *ABL*, *BCR* or *GUSB*. For each dataset, 2 sets of ERM-AD623 were used to produce multiple calibration curves in different qPCR runs and on different days. In addition, the labs were also asked to measure the copy numbers of *BCR-ABL1* (e14a2), the copy numbers of the CG and the copy number ratio *BCR-ABL1/CG* from 2 cDNA samples using ERM-AD623 as a calibrator. cDNA samples were prepared from the K562 cell line following the standardised EAC reverse transcription protocol.[^5^](#_ENREF_5) The cDNA stock solution was diluted to 2 different concentrations with estimated *BCR-ABL1* e14a2 transcript copy numbers of 3.60 x 10^4^ ± 0.30 x 10^4^ cp/µL and 1.30 x 10^3^ ± 0.04 x 10^3^ cp/µL (measured by digital PCR, the mean value and standard deviation from five panels on one digital array).

Armored RNA (aRNAs) for *BCR-ABL1* and *ABL1* have been described previously[^11^](#_ENREF_11) and were obtained from Asuragen Inc. (Austin, TX, USA). Mixtures of K562 (*BCR-ABL1* positive) and HL60 (*BCR-ABL1* negative) cells were made as described and lysed in Trizol (Invitrogen, Paisley, UK) or RLT Plus (Qiagen, Manchester, UK). Mixtures were kept frozen at -70^o^C and distributed to laboratories on dry ice.

In the large scale study, two different levels of e14a2 aRNA diluted in a background of *ABL1* aRNA predicted to correspond to MMR (0.1% *BCR-ABL1* / *ABL1*) and MR^4^ (0.01% *BCR-ABL1* / *ABL1*) and three different cell line lysates (approximately 5%, 0.05%, 0.005% *BCR-ABL1*^IS^) were sent to 57 laboratories for analysis. Laboratories were asked to report copy numbers of *BCR-ABL1* (e14a2/b3a2), copy numbers of *ABL1* and the *BCR-ABL1* / *ABL1* ratios before and after conversion to the international scale (IS) using ERM-AD623 as a common plasmid standard curve and their usual laboratory plasmid standard curve.

**SUPPLEMENTARY FIGURES:**

**Suppl. Fig 1. Alignment of the results obtained by sequence analysis and the published consensus sequences**

The sequences of the fragments were determined in two independent laboratories (Lab1 and Lab 2) and were compared with the consensus sequences of the transcripts of *GUSB*, *BCR* and *ABL* available in the GenBank database (NCBI, NIH, Bethesda, MD, US). The following consensus sequences were used: NM_00181.2 (gi119372313) from nucleotide 1216 to 2028 for the *GUSB* transcript, NM_004327.3 (gi82546842) from nucleotide 3345 to 4307 for *BCR* and NM_004327.3 (gi82546842) from nucleotide 2800 to 3377 combined with NM-005157.3 (gi62362413) from nucleotide 83 to 901 for *BCR-ABL1*. The alignment of these sequences shows that the three fragments are present at single copy in the plasmid.

Two differences were observed between the consensus sequences from Genbank and the sequences of the fragments present in pIRMM-0099. These differences correspond to two known single nucleotide polymorphisms (SNPs) present in the SNP database (dbSNP, NCBI, http://www.ncbi.nlm.nih.gov/projects/SNP). The DNA fragment of the *BCR-ABL* transcript contains the G allele of the SNP with reference number rs140504 while the consensus sequence in the NCBI database contains the A allele. Within the DNA fragment of the *BCR* transcript, the T allele of SNP rs11558697 is present while in the consensus sequence the C allele is present. The presence of these SNPs should be taken in account when using primers or probes located at these positions.

Lab1 1 atctgcagaattcggcttgtaaacgggctgttttccaaacattgtgacttggctactgagtggggatacctggtttcatt

Lab2 1 atctgcagaattcggcttgtaaacgggctgttttccaaacattgtgacttggctactgagtggggatacctggtttcatt

GUSB 813 ------------------gtaaacgggctgttttccaaacattgtgacttggctactgagtggggatacctggtttcatt

BCR --------------------------------------------------------------------------------

BCR-ABL1 --------------------------------------------------------------------------------

Lab1 81 ggcaatcttccagtatctctctcgcaaaaggaacgctgcactttttggttgtctctgccgagtgaagatcccctttttat

Lab2 81 ggcaatcttccagtatctctctcgcaaaaggaacgctgcactttttggttgtctctgccgagtgaagatcccctttttat

GUSB 751 ggcaatcttccagtatctctctcgcaaaaggaacgctgcactttttggttgtctctgccgagtgaagatcccctttttat

BCR --------------------------------------------------------------------------------

BCR-ABL1 --------------------------------------------------------------------------------

Lab1 161 tccccagcactctcgtcggtgactgttcagtcatgaaatcggcaaaattccaaatgagctctccaaccacgtattttctg

Lab2 161 tccccagcactctcgtcggtgactgttcagtcatgaaatcggcaaaattccaaatgagctctccaaccacgtattttctg

GUSB 671 tccccagcactctcgtcggtgactgttcagtcatgaaatcggcaaaattccaaatgagctctccaaccacgtattttctg

BCR --------------------------------------------------------------------------------

BCR-ABL1 --------------------------------------------------------------------------------

Lab1 241 cgtttttgatccagacccagatggtactgctctagcagacttttctggtactcttcagtgaacatcagaggtggatcctg

Lab2 241 cgtttttgatccagacccagatggtactgctctagcagacttttctggtactcttcagtgaacatcagaggtggatcctg

GUSB 591 cgtttttgatccagacccagatggtactgctctagcagacttttctggtactcttcagtgaacatcagaggtggatcctg

BCR --------------------------------------------------------------------------------

BCR-ABL1 --------------------------------------------------------------------------------

Lab1 321 gtgaaaccctgcaatcgtttctgctccatactcgctctgaataatgggcttctgatacttcttataccagttctcaaact

Lab2 321 gtgaaaccctgcaatcgtttctgctccatactcgctctgaataatgggcttctgatacttcttataccagttctcaaact

GUSB 511 gtgaaaccctgcaatcgtttctgctccatactcgctctgaataatgggcttctgatacttcttataccagttctcaaact

BCR --------------------------------------------------------------------------------

BCR-ABL1 --------------------------------------------------------------------------------

Lab1 401 gggtggccagctgcagctgaatcaactccaggtgcccgtagtcgtgataccaagagtagtagctgttcaaacagatcaca

Lab2 401 gggtggccagctgcagctgaatcaactccaggtgcccgtagtcgtgataccaagagtagtagctgttcaaacagatcaca

GUSB 431 gggtggccagctgcagctgaatcaactccaggtgcccgtagtcgtgataccaagagtagtagctgttcaaacagatcaca

BCR --------------------------------------------------------------------------------

BCR-ABL1 --------------------------------------------------------------------------------

Lab1 481 tccacatacggagcccccttgtctgctgcatagttagagttgctcacaaaggtcacaggccgggaggggtccaaggattt

Lab2 481 tccacatacggagcccccttgtctgctgcatagttagagttgctcacaaaggtcacaggccgggaggggtccaaggattt

GUSB 351 tccacatacggagcccccttgtctgctgcatagttagagttgctcacaaaggtcacaggccgggaggggtccaaggattt

BCR --------------------------------------------------------------------------------

BCR-ABL1 --------------------------------------------------------------------------------

Lab1 561 ggtgtgagcgatcaccatcttcaagtagtagccagcagattctaggtgggacgcaggctcgttggccacagaccacatca

Lab2 561 ggtgtgagcgatcaccatcttcaagtagtagccagcagattctaggtgggacgcaggctcgttggccacagaccacatca

GUSB 271 ggtgtgagcgatcaccatcttcaagtagtagccagcagattctaggtgggacgcaggctcgttggccacagaccacatca

BCR --------------------------------------------------------------------------------

BCR-ABL1 --------------------------------------------------------------------------------

Lab1 641 cgaccgcggggtggttcttgtccctacgcaccacttcttccatcacctgcatgtggtgatgcagagaaacgttgttgaag

Lab2 641 cgaccgcggggtggttcttgtccctacgcaccacttcttccatcacctgcatgtggtgatgcagagaaacgttgttgaag

GUSB 191 cgaccgcggggtggttcttgtccctacgcaccacttcttccatcacctgcatgtggtgatgcagagaaacgttgttgaag

BCR --------------------------------------------------------------------------------

BCR-ABL1 --------------------------------------------------------------------------------

Lab1 721 aactgcggcagcgccaggcccacgccgggacactcatcgatgaccacaatcccatagcggtcacacatctgcatcacttc

Lab2 721 aactgcggcagcgccaggcccacgccgggacactcatcgatgaccacaatcccatagcggtcacacatctgcatcacttc

GUSB 111 aactgcggcagcgccaggcccacgccgggacactcatcgatgaccacaatcccatagcggtcacacatctgcatcacttc

BCR --------------------------------------------------------------------------------

BCR-ABL1 --------------------------------------------------------------------------------

Lab1 801 ctctgcataggggtagtggctggtacggaaaaagccgaattccagcacactggcggccgttactagtggatccgagctcg

Lab2 801 ctctgcataggggtagtggctggtacggaaaaagccgaattccagcacactggcggccgttactagtggatccgagctcg

GUSB 31 ctctgcataggggtagtggctggtacggaaa-------------------------------------------------

BCR --------------------------------------------------------------------------------

BCR-ABL1 --------------------------------------------------------------------------------

Lab1 881 gtaccgagctcgaattcggcttgtccactcagccactggatttaagcagagttcaaatctgtactgcaccctggaggtgg

Lab2 881 gtaccgagctcgaattcggcttgtccactcagccactggatttaagcagagttcaaatctgtactgcaccctggaggtgg

GUSB --------------------------------------------------------------------------------

BCR 1 ----------------------gtccactcagccactggatttaagcagagttcaaatctgtactgcaccctggaggtgg

BCR-ABL1 --------------------------------------------------------------------------------

Lab1 961 attcctttgggtattttgtgaataaagcaaagacgcgcgtctacagggacacagctgagccaaactggaacgaggaattt

Lab2 961 attcctttgggtattttgtgaataaagcaaagacgcgcgtctacagggacacagctgagccaaactggaacgaggaattt

GUSB --------------------------------------------------------------------------------

BCR 59 attcctttgggtattttgtgaataaagcaaagacgcgcgtctacagggacacagctgagccaaactggaacgaggaattt

BCR-ABL1 --------------------------------------------------------------------------------

Lab1 1041 gagatagagctggagggctcccagaccctgaggatactgtgctatgaaaagtgttacaacaagacgaagatccccaagga

Lab2 1041 gagatagagctggagggctcccagaccctgaggatactgtgctatgaaaagtgttacaacaagacgaagatccccaagga

GUSB --------------------------------------------------------------------------------

BCR 139 gagatagagctggagggctcccagaccctgaggatactgtgctatgaaaagtgttacaacaagacgaagatccccaagga

BCR-ABL1 --------------------------------------------------------------------------------

Lab1 1121 ggacggcgagagcacggacagactcatggggaagggccaggtccagctggacccgcaggccctgcaggacagagactggc

Lab2 1121 ggacggcgagagcacggacagactcatggggaagggccaggtccagctggacccgcaggccctgcaggacagagactggc

GUSB --------------------------------------------------------------------------------

BCR 219 ggacggcgagagcacggacagactcatggggaagggccaggtccagctggacccgcaggccctgcaggacagagactggc

BCR-ABL1 --------------------------------------------------------------------------------

Lab1 1201 agcgcaccgtcatcgccatgaatgggatcgaagtaaagctctcggtcaagttcaacagcagggagttcagcttgaagagg

Lab2 1201 agcgcaccgtcatcgccatgaatgggatcgaagtaaagctctcggtcaagttcaacagcagggagttcagcttgaagagg

GUSB --------------------------------------------------------------------------------

BCR 299 agcgcaccgtcatcgccatgaatgggatcgaagtaaagctctcggtcaagttcaacagcagggagttcagcttgaagagg

BCR-ABL1 --------------------------------------------------------------------------------

Lab1 1281 atgccgtcccgaaaacagacaggggtcttcggagtcaagattgctgtggtcaccaagagagagaggtccaaggtgcccta

Lab2 1281 atgccgtcccgaaaacagacaggggtcttcggagtcaagattgctgtggtcaccaagagagagaggtccaaggtgcccta

GUSB --------------------------------------------------------------------------------

BCR 379 atgccgtcccgaaaacagacaggggtcttcggagtcaagattgctgtggtcaccaagagagagaggtccaaggtgcccta

BCR-ABL1 --------------------------------------------------------------------------------

Lab1 1361 catcgtgcgccagtgcgtggaggagatcgagcgccgaggcatggaggaggtgggcatctaccgcgtgtccggtgtggcca

Lab2 1361 catcgtgcgccagtgcgtggaggagatcgagcgccgaggcatggaggaggtgggcatctaccgcgtgtccggtgtggcca

GUSB --------------------------------------------------------------------------------

BCR 459 catcgtgcgccagtgcgtggaggagatcgagcgccgaggcatggaggaggtgggcatctaccgcgtgtccggtgtggcca

BCR-ABL1 --------------------------------------------------------------------------------

Lab1 1441 cggacatccaggcactgaaggcagccttcgacgtcaataacaaggatgtgtcggtgatgatgagcgagatggacgtgaac

Lab2 1441 cggacatccaggcactgaaggcagccttcgacgtcaataacaaggatgtgtcggtgatgatgagcgagatggacgtgaac

GUSB --------------------------------------------------------------------------------

BCR 539 cggacatccaggcactgaaggcagccttcgacgtcaataacaaggacgtgtcggtgatgatgagcgagatggacgtgaac

BCR-ABL1 --------------------------------------------------------------------------------

Lab1 1521 gccatcgcaggcacgctgaagctgtacttccgtgagctgcccgagcccctcttcactgacgagttctaccccaacttcgc

Lab2 1521 gccatcgcaggcacgctgaagctgtacttccgtgagctgcccgagcccctcttcactgacgagttctaccccaacttcgc

GUSB --------------------------------------------------------------------------------

BCR 619 gccatcgcaggcacgctgaagctgtacttccgtgagctgcccgagcccctcttcactgacgagttctaccccaacttcgc

BCR-ABL1 --------------------------------------------------------------------------------

Lab1 1601 agagggcatcgctctttcagacccggttgcaaaggagagctgcatgctcaacctgctgctgtccctgccggaggccaacc

Lab2 1601 agagggcatcgctctttcagacccggttgcaaaggagagctgcatgctcaacctgctgctgtccctgccggaggccaacc

GUSB --------------------------------------------------------------------------------

BCR 699 agagggcatcgctctttcagacccggttgcaaaggagagctgcatgctcaacctgctgctgtccctgccggaggccaacc

BCR-ABL1 --------------------------------------------------------------------------------

Lab1 1681 tgctcaccttccttttccttctggaccacctgaaaagggtggcagagaaggaggcagtcaataagatgtccctgcacaac

Lab2 1681 tgctcaccttccttttccttctggaccacctgaaaagggtggcagagaaggaggcagtcaataagatgtccctgcacaac

GUSB --------------------------------------------------------------------------------

BCR 779 tgctcaccttccttttccttctggaccacctgaaaagggtggcagagaaggaggcagtcaataagatgtccctgcacaac

BCR-ABL1 --------------------------------------------------------------------------------

Lab1 1761 ctcgccacggtctttggccccacgctgctccggccctccgagaaggagagcaagctccctgccaaccccagccagcctat

Lab2 1761 ctcgccacggtctttggccccacgctgctccggccctccgagaaggagagcaagctccctgccaaccccagccagcctat

GUSB --------------------------------------------------------------------------------

BCR 859 ctcgccacggtctttggccccacgctgctccggccctccgagaaggagagcaagctccctgccaaccccagccagcctat

BCR-ABL1 --------------------------------------------------------------------------------

Lab1 1841 caccatgactgacagctggtccttgaagccgaattcgtaatcatgtcatagctgtttcctgtgtgaaattgttatccgct

Lab2 1841 caccatgactgacagctggtccttgaagccgaattcgtaatcatgtcatagctgtttcctgtgtgaaattgttatccgct

GUSB --------------------------------------------------------------------------------

BCR 939 caccatgactgacagctggtccttg-------------------------------------------------------

BCR-ABL1 --------------------------------------------------------------------------------

Lab1 1921 cacaattccacacaacatacgagccggaagcataaagtgtaaagcctggggtgcctaatgagtgagctaactcacattaa

Lab2 1921 cacaattccacacaacatacgagccggaagcataaagtgtaaagcctggggtgcctaatgagtgagctaactcacattaa

GUSB --------------------------------------------------------------------------------

BCR --------------------------------------------------------------------------------

BCR-ABL1 --------------------------------------------------------------------------------

Lab1 2001 ttgcgttgcgctcactgcccgctttccagtcgggaaacctgtcgtgccagctgcattaatgaatcggccaacgcgcgggg

Lab2 2001 ttgcgttgcgctcactgcccgctttccagtcgggaaacctgtcgtgccagctgcattaatgaatcggccaacgcgcgggg

GUSB --------------------------------------------------------------------------------

BCR --------------------------------------------------------------------------------

BCR-ABL1 --------------------------------------------------------------------------------

Lab1 2081 agaggcggtttgcgtattgggcgctcttccgcttcctcgctcactgactcgctgcgctcggtcgttcggctgcggcgagc

Lab2 2081 agaggcggtttgcgtattgggcgctcttccgcttcctcgctcactgactcgctgcgctcggtcgttcggctgcggcgagc

GUSB --------------------------------------------------------------------------------

BCR --------------------------------------------------------------------------------

BCR-ABL1 --------------------------------------------------------------------------------

Lab1 2161 ggtatcagctcactcaaaggcggtaatacggttatccacagaatcaggggataacgcaggaaagaacatgtgagcaaaag

Lab2 2161 ggtatcagctcactcaaaggcggtaatacggttatccacagaatcaggggataacgcaggaaagaacatgtgagcaaaag

GUSB --------------------------------------------------------------------------------

BCR --------------------------------------------------------------------------------

BCR-ABL1 --------------------------------------------------------------------------------

Lab1 2241 gccagcaaaaggccaggaaccgtaaaaaggccgcgttgctggcgtttttccataggctccgcccccctgacgagcatcac

Lab2 2241 gccagcaaaaggccaggaaccgtaaaaaggccgcgttgctggcgtttttccataggctccgcccccctgacgagcatcac

GUSB --------------------------------------------------------------------------------

BCR --------------------------------------------------------------------------------

BCR-ABL1 --------------------------------------------------------------------------------

Lab1 2321 aaaaatcgacgctcaagtcagaggtggcgaaacccgacaggactataaagataccaggcgtttccccctggaagctccct

Lab2 2321 aaaaatcgacgctcaagtcagaggtggcgaaacccgacaggactataaagataccaggcgtttccccctggaagctccct

GUSB --------------------------------------------------------------------------------

BCR --------------------------------------------------------------------------------

BCR-ABL1 --------------------------------------------------------------------------------

Lab1 2401 cgtgcgctctcctgttccgaccctgccgcttaccggatacctgtccgcctttctcccttcgggaagcgtggcgctttctc

Lab2 2401 cgtgcgctctcctgttccgaccctgccgcttaccggatacctgtccgcctttctcccttcgggaagcgtggcgctttctc

GUSB --------------------------------------------------------------------------------

BCR --------------------------------------------------------------------------------

BCR-ABL1 --------------------------------------------------------------------------------

Lab1 2481 atagctcacgctgtaggtatctcagttcggtgtaggtcgttcgctccaagctgggctgtgtgcacgaaccccccgttcag

Lab2 2481 atagctcacgctgtaggtatctcagttcggtgtaggtcgttcgctccaagctgggctgtgtgcacgaaccccccgttcag

GUSB --------------------------------------------------------------------------------

BCR --------------------------------------------------------------------------------

BCR-ABL1 --------------------------------------------------------------------------------

Lab1 2561 cccgaccgctgcgccttatccggtaactatcgtcttgagtccaacccggtaagacacgacttatcgccactggcagcagc

Lab2 2561 cccgaccgctgcgccttatccggtaactatcgtcttgagtccaacccggtaagacacgacttatcgccactggcagcagc

GUSB --------------------------------------------------------------------------------

BCR --------------------------------------------------------------------------------

BCR-ABL1 --------------------------------------------------------------------------------

Lab1 2641 cactggtaacaggattagcagagcgaggtatgtaggcggtgctacagagttcttgaagtggtggcctaactacggctaca

Lab2 2641 cactggtaacaggattagcagagcgaggtatgtaggcggtgctacagagttcttgaagtggtggcctaactacggctaca

GUSB --------------------------------------------------------------------------------

BCR --------------------------------------------------------------------------------

BCR-ABL1 --------------------------------------------------------------------------------

Lab1 2721 ctagaagaacagtatttggtatctgcgctctgctgaagccagttaccttcggaaaaagagttggtagctcttgatccggc

Lab2 2721 ctagaagaacagtatttggtatctgcgctctgctgaagccagttaccttcggaaaaagagttggtagctcttgatccggc

GUSB --------------------------------------------------------------------------------

BCR --------------------------------------------------------------------------------

BCR-ABL1 --------------------------------------------------------------------------------

Lab1 2801 aaacaaaccaccgctggtagcggtggtttttttgtttgcaagcagcagattacgcgcagaaaaaaaggatctcaagaaga

Lab2 2801 aaacaaaccaccgctggtagcggtggtttttttgtttgcaagcagcagattacgcgcagaaaaaaaggatctcaagaaga

GUSB --------------------------------------------------------------------------------

BCR --------------------------------------------------------------------------------

BCR-ABL1 --------------------------------------------------------------------------------

Lab1 2881 tcctttgatcttttctacggggtctgacgctcagtggaacgaaaactcacgttaagggattttggtcatgagattatcaa

Lab2 2881 tcctttgatcttttctacggggtctgacgctcagtggaacgaaaactcacgttaagggattttggtcatgagattatcaa

GUSB --------------------------------------------------------------------------------

BCR --------------------------------------------------------------------------------

BCR-ABL1 --------------------------------------------------------------------------------

Lab1 2961 aaaggatcttcacctagatccttttaaattaaaaatgaagttttaaatcaatctaaagtatatatgagtaaacttggtct

Lab2 2961 aaaggatcttcacctagatccttttaaattaaaaatgaagttttaaatcaatctaaagtatatatgagtaaacttggtct

GUSB --------------------------------------------------------------------------------

BCR --------------------------------------------------------------------------------

BCR-ABL1 --------------------------------------------------------------------------------

Lab1 3041 gacagttaccaatgcttaatcagtgaggcacctatctcagcgatctgtctatttcgttcatccatagttgcctgactccc

Lab2 3041 gacagttaccaatgcttaatcagtgaggcacctatctcagcgatctgtctatttcgttcatccatagttgcctgactccc

GUSB --------------------------------------------------------------------------------

BCR --------------------------------------------------------------------------------

BCR-ABL1 --------------------------------------------------------------------------------

Lab1 3121 cgtcgtgtagataactacgatacgggagggcttaccatctggccccagtgctgcaatgataccgcgagacccacgctcac

Lab2 3121 cgtcgtgtagataactacgatacgggagggcttaccatctggccccagtgctgcaatgataccgcgagacccacgctcac

GUSB --------------------------------------------------------------------------------

BCR --------------------------------------------------------------------------------

BCR-ABL1 --------------------------------------------------------------------------------

Lab1 3201 cggctccagatttatcagcaataaaccagccagccggaagggccgagcgcagaagtggtcctgcaactttatccgcctcc

Lab2 3201 cggctccagatttatcagcaataaaccagccagccggaagggccgagcgcagaagtggtcctgcaactttatccgcctcc

GUSB --------------------------------------------------------------------------------

BCR --------------------------------------------------------------------------------

BCR-ABL1 --------------------------------------------------------------------------------

Lab1 3281 atccagtctattaattgttgccgggaagctagagtaagtagttcgccagttaatagtttgcgcaacgttgttgccattgc

Lab2 3281 atccagtctattaattgttgccgggaagctagagtaagtagttcgccagttaatagtttgcgcaacgttgttgccattgc

GUSB --------------------------------------------------------------------------------

BCR --------------------------------------------------------------------------------

BCR-ABL1 --------------------------------------------------------------------------------

Lab1 3361 tacaggcatcgtggtgtcacgctcgtcgtttggtatggcttcattcagctccggttcccaacgatcaaggcgagttacat

Lab2 3361 tacaggcatcgtggtgtcacgctcgtcgtttggtatggcttcattcagctccggttcccaacgatcaaggcgagttacat

GUSB --------------------------------------------------------------------------------

BCR --------------------------------------------------------------------------------

BCR-ABL1 --------------------------------------------------------------------------------

Lab1 3441 gatcccccatgttgtgcaaaaaagcggttagctccttcggtcctccgatcgttgtcagaagtaagttggccgcagtgtta

Lab2 3441 gatcccccatgttgtgcaaaaaagcggttagctccttcggtcctccgatcgttgtcagaagtaagttggccgcagtgtta

GUSB --------------------------------------------------------------------------------

BCR --------------------------------------------------------------------------------

BCR-ABL1 --------------------------------------------------------------------------------

Lab1 3521 tcactcatggttatggcagcactgcataattctcttactgtcatgccatccgtaagatgcttttctgtgactggtgagta

Lab2 3521 tcactcatggttatggcagcactgcataattctcttactgtcatgccatccgtaagatgcttttctgtgactggtgagta

GUSB --------------------------------------------------------------------------------

BCR --------------------------------------------------------------------------------

BCR-ABL1 --------------------------------------------------------------------------------

Lab1 3601 ctcaaccaagtcattctgagaatagtgtatgcggcgaccgagttgctcttgcccggcgtcaatacgggataataccgcgc

Lab2 3601 ctcaaccaagtcattctgagaatagtgtatgcggcgaccgagttgctcttgcccggcgtcaatacgggataataccgcgc

GUSB --------------------------------------------------------------------------------

BCR --------------------------------------------------------------------------------

BCR-ABL1 --------------------------------------------------------------------------------

Lab1 3681 cacatagcagaactttaaaagtgctcatcattggaaaacgttcttcggggcgaaaactctcaaggatcttaccgctgttg

Lab2 3681 cacatagcagaactttaaaagtgctcatcattggaaaacgttcttcggggcgaaaactctcaaggatcttaccgctgttg

GUSB --------------------------------------------------------------------------------

BCR --------------------------------------------------------------------------------

BCR-ABL1 --------------------------------------------------------------------------------

Lab1 3761 agatccagttcgatgtaacccactcgtgcacccaactgatcttcagcatcttttactttcaccagcgtttctgggtgagc

Lab2 3761 agatccagttcgatgtaacccactcgtgcacccaactgatcttcagcatcttttactttcaccagcgtttctgggtgagc

GUSB --------------------------------------------------------------------------------

BCR --------------------------------------------------------------------------------

BCR-ABL1 --------------------------------------------------------------------------------

Lab1 3841 aaaaacaggaaggcaaaatgccgcaaaaaagggaataagggcgacacggaaatgttgaatactcatactcttcctttttc

Lab2 3841 aaaaacaggaaggcaaaatgccgcaaaaaagggaataagggcgacacggaaatgttgaatactcatactcttcctttttc

GUSB --------------------------------------------------------------------------------

BCR --------------------------------------------------------------------------------

BCR-ABL1 --------------------------------------------------------------------------------

Lab1 3921 aatattattgaagcatttatcagggttattgtctcatgagcggatacatatttgaatgtatttagaaaaataaacaaata

Lab2 3921 aatattattgaagcatttatcagggttattgtctcatgagcggatacatatttgaatgtatttagaaaaataaacaaata

GUSB --------------------------------------------------------------------------------

BCR --------------------------------------------------------------------------------

BCR-ABL1 --------------------------------------------------------------------------------

Lab1 4001 ggggttccgcgcacatttccccgaaaagtgccacctgacgtctaagaaaccattattatcatgacattaacctataaaaa

Lab2 4001 ggggttccgcgcacatttccccgaaaagtgccacctgacgtctaagaaaccattattatcatgacattaacctataaaaa

GUSB --------------------------------------------------------------------------------

BCR --------------------------------------------------------------------------------

BCR-ABL1 --------------------------------------------------------------------------------

Lab1 4081 taggcgtatcacgaggccctttcgtctcgcgcgtttcggtgatgacggtgaaaacctctgacacatgcagctcccggaga

Lab2 4081 taggcgtatcacgaggccctttcgtctcgcgcgtttcggtgatgacggtgaaaacctctgacacatgcagctcccggaga

GUSB --------------------------------------------------------------------------------

BCR --------------------------------------------------------------------------------

BCR-ABL1 --------------------------------------------------------------------------------

Lab1 4161 cggtcacagcttgtctgtaagcggatgccgggagcagacaagcccgtcagggcgcgtcagcgggtgttggcgggtgtcgg

Lab2 4161 cggtcacagcttgtctgtaagcggatgccgggagcagacaagcccgtcagggcgcgtcagcgggtgttggcgggtgtcgg

GUSB --------------------------------------------------------------------------------

BCR --------------------------------------------------------------------------------

Lab1 4241 ggctggcttaactatgcggcatcagagcagattgtactgagagtgcaccatatgcggtgtgaaataccgcacagatgcgt

Lab2 4241 ggctggcttaactatgcggcatcagagcagattgtactgagagtgcaccatatgcggtgtgaaataccgcacagatgcgt

GUSB --------------------------------------------------------------------------------

BCR --------------------------------------------------------------------------------

BCR-ABL1 --------------------------------------------------------------------------------

Lab1 4321 aaggagaaaataccgcatcaggcgccattcgccattcaggctgcgcaactgttgggaagggcgatcggtgcgggcctctt

Lab2 4321 aaggagaaaataccgcatcaggcgccattcgccattcaggctgcgcaactgttgggaagggcgatcggtgcgggcctctt

GUSB --------------------------------------------------------------------------------

BCR --------------------------------------------------------------------------------

BCR-ABL1 --------------------------------------------------------------------------------

Lab1 4401 cgctattacgccagctggcgaaagggggatgtgctgcaaggcgattaagttgggtaacgccagggttttcccagtcacga

Lab2 4401 cgctattacgccagctggcgaaagggggatgtgctgcaaggcgattaagttgggtaacgccagggttttcccagtcacga

GUSB --------------------------------------------------------------------------------

BCR --------------------------------------------------------------------------------

BCR-ABL1 --------------------------------------------------------------------------------

Lab1 4481 cgttgtaaaacgacggccagtgccaagcttgcatgcctgcaggtcgactctctgcaccaagctcaagaagcagagcggag

Lab2 4481 cgttgtaaaacgacggccagtgccaagcttgcatgcctgcaggtcgactctctgcaccaagctcaagaagcagagcggag

GUSB --------------------------------------------------------------------------------

BCR --------------------------------------------------------------------------------

BCR-ABL1 1 ------------------------------------------------tctctgcaccaagctcaagaagcagagcggag

Lab1 4561 gcaaaacgcagcagtatgactgcaaatggtacattccgctcacggatctcagcttccagatggtggatgaactggaggca

Lab2 4561 gcaaaacgcagcagtatgactgcaaatggtacattccgctcacggatctcagcttccagatggtggatgaactggaggca

GUSB --------------------------------------------------------------------------------

BCR --------------------------------------------------------------------------------

BCR-ABL1 33 gcaaaacgcagcagtatgactgcaaatggtacattccgctcacggatctcagcttccagatggtggatgaactggaggca

Lab1 4641 gtgcccaacatccccctggtgcccgatgaggagctggacgctttgaagatcaagatctcccagatcaagagtgacatcca

Lab2 4641 gtgcccaacatccccctggtgcccgatgaggagctggacgctttgaagatcaagatctcccagatcaagagtgacatcca

GUSB --------------------------------------------------------------------------------

BCR --------------------------------------------------------------------------------

BCR-ABL1 113 gtgcccaacatccccctggtgcccgatgaggagctggacgctttgaagatcaagatctcccagatcaagaatgacatcca

Lab1 4721 gagagagaagagggcgaacaagggcagcaaggctacggagaggctgaagaagaagctgtcggagcaggagtcactgctgc

Lab2 4721 gagagagaagagggcgaacaagggcagcaaggctacggagaggctgaagaagaagctgtcggagcaggagtcactgctgc

GUSB --------------------------------------------------------------------------------

BCR --------------------------------------------------------------------------------

BCR-ABL1 193 gagagagaagagggcgaacaagggcagcaaggctacggagaggctgaagaagaagctgtcggagcaggagtcactgctgc

Lab1 4801 tgcttatgtctcccagcatggccttcagggtgcacagccgcaacggcaagagttacacgttcctgatctcctctgactat

Lab2 4801 tgcttatgtctcccagcatggccttcagggtgcacagccgcaacggcaagagttacacgttcctgatctcctctgactat

GUSB --------------------------------------------------------------------------------

BCR --------------------------------------------------------------------------------

BCR-ABL1 273 tgcttatgtctcccagcatggccttcagggtgcacagccgcaacggcaagagttacacgttcctgatctcctctgactat

Lab1 4881 gagcgtgcagagtggagggagaacatccgggagcagcagaagaagtgtttcagaagcttctccctgacatccgtggagct

Lab2 4881 gagcgtgcagagtggagggagaacatccgggagcagcagaagaagtgtttcagaagcttctccctgacatccgtggagct

GUSB --------------------------------------------------------------------------------

BCR --------------------------------------------------------------------------------

BCR-ABL1 353 gagcgtgcagagtggagggagaacatccgggagcagcagaagaagtgtttcagaagcttctccctgacatccgtggagct

Lab1 4961 gcagatgctgaccaactcgtgtgtgaaactccagactgtccacagcattccgctgaccatcaataaggaagatgatgagt

Lab2 4961 gcagatgctgaccaactcgtgtgtgaaactccagactgtccacagcattccgctgaccatcaataaggaagatgatgagt

GUSB --------------------------------------------------------------------------------

BCR --------------------------------------------------------------------------------

BCR-ABL1 433 gcagatgctgaccaactcgtgtgtgaaactccagactgtccacagcattccgctgaccatcaataaggaagatgatgagt

Lab1 5041 ctccggggctctatgggtttctgaatgtcatcgtccactcagccactggatttaagcagagttcaaaagcccttcagcgg

Lab2 5041 ctccggggctctatgggtttctgaatgtcatcgtccactcagccactggatttaagcagagttcaaaagcccttcagcgg

GUSB --------------------------------------------------------------------------------

BCR --------------------------------------------------------------------------------

BCR-ABL1 513 ctccggggctctatgggtttctgaatgtcatcgtccactcagccactggatttaagcagagttcaaaagcccttcagcgg

Lab1 5121 ccagtagcatctgactttgagcctcagggtctgagtgaagccgctcgttggaactccaaggaaaaccttctcgctggacc

Lab2 5121 ccagtagcatctgactttgagcctcagggtctgagtgaagccgctcgttggaactccaaggaaaaccttctcgctggacc

GUSB --------------------------------------------------------------------------------

BCR --------------------------------------------------------------------------------

BCR-ABL1 593 ccagtagcatctgactttgagcctcagggtctgagtgaagccgctcgttggaactccaaggaaaaccttctcgctggacc

Lab1 5201 cagtgaaaatgaccccaaccttttcgttgcactgtatgattttgtggccagtggagataacactctaagcataactaaag

Lab2 5201 cagtgaaaatgaccccaaccttttcgttgcactgtatgattttgtggccagtggagataacactctaagcataactaaag

GUSB --------------------------------------------------------------------------------

BCR --------------------------------------------------------------------------------

BCR-ABL1 673 cagtgaaaatgaccccaaccttttcgttgcactgtatgattttgtggccagtggagataacactctaagcataactaaag

Lab1 5281 gtgaaaagctccgggtcttaggctataatcacaatggggaatggtgtgaagcccaaaccaaaaatggccaaggctgggtc

Lab2 5281 gtgaaaagctccgggtcttaggctataatcacaatggggaatggtgtgaagcccaaaccaaaaatggccaaggctgggtc

GUSB --------------------------------------------------------------------------------

BCR --------------------------------------------------------------------------------

BCR-ABL1 753 gtgaaaagctccgggtcttaggctataatcacaatggggaatggtgtgaagcccaaaccaaaaatggccaaggctgggtc

Lab1 5361 ccaagcaactacatcacgccagtcaacagtctggagaaacactcctggtaccatgggcctgtgtcccgcaatgccgctga

Lab2 5361 ccaagcaactacatcacgccagtcaacagtctggagaaacactcctggtaccatgggcctgtgtcccgcaatgccgctga

GUSB --------------------------------------------------------------------------------

BCR --------------------------------------------------------------------------------

BCR-ABL1 833 ccaagcaactacatcacgccagtcaacagtctggagaaacactcctggtaccatgggcctgtgtcccgcaatgccgctga

Lab1 5441 gtatctgctgagcagcgggatcaatggcagcttcttggtgcgtgagagtgagagcagtcctggccagaggtccatctcgc

Lab2 5441 gtatctgctgagcagcgggatcaatggcagcttcttggtgcgtgagagtgagagcagtcctggccagaggtccatctcgc

GUSB --------------------------------------------------------------------------------

BCR --------------------------------------------------------------------------------

BCR-ABL1 913 gtatctgctgagcagcgggatcaatggcagcttcttggtgcgtgagagtgagagcagtcctggccagaggtccatctcgc

Lab1 5521 tgagatacgaagggagggtgtaccattacaggatcaacactgcttctgatggcaagctctacgtctcctccgagagccgc

Lab2 5521 tgagatacgaagggagggtgtaccattacaggatcaacactgcttctgatggcaagctctacgtctcctccgagagccgc

GUSB --------------------------------------------------------------------------------

BCR --------------------------------------------------------------------------------

BCR-ABL1 993 tgagatacgaagggagggtgtaccattacaggatcaacactgcttctgatggcaagctctacgtctcctccgagagccgc

Lab1 5601 ttcaacaccctggccgagttggttcatcatcattcaacggtggccgacgggctcatcaccacgctccattatccagcccc

Lab2 5601 ttcaacaccctggccgagttggttcatcatcattcaacggtggccgacgggctcatcaccacgctccattatccagcccc

GUSB --------------------------------------------------------------------------------

BCR --------------------------------------------------------------------------------

BCR-ABL1 1073 ttcaacaccctggccgagttggttcatcatcattcaacggtggccgacgggctcatcaccacgctccattatccagcccc

Lab1 5681 aaagcgcaacaagcccactgtctatggtgtgtcccccaactacgacaagtgggagatggaacgcacggacatcaccatga

Lab2 5681 aaagcgcaacaagcccactgtctatggtgtgtcccccaactacgacaagtgggagatggaacgcacggacatcaccatga

GUSB --------------------------------------------------------------------------------

BCR --------------------------------------------------------------------------------

BCR-ABL1 1153 aaagcgcaacaagcccactgtctatggtgtgtcccccaactacgacaagtgggagatggaacgcacggacatcaccatga

Lab1 5761 agcacaagctgggcgggggccagtacggggaggtgtacgagggcgtgtggaagaaatacagcctgacggtggccgtgaag

Lab2 5761 agcacaagctgggcgggggccagtacggggaggtgtacgagggcgtgtggaagaaatacagcctgacggtggccgtgaag

GUSB --------------------------------------------------------------------------------

BCR --------------------------------------------------------------------------------

BCR-ABL1 1233 agcacaagctgggcgggggccagtacggggaggtgtacgagggcgtgtggaagaaatacagcctgacggtggccgtgaag

Lab1 5841 accttgaaggaggacaccatggaggtggaagagttcttgaaagaagctgcagtcatgaaagagatcaaacaccctaacct

Lab2 5841 accttgaaggaggacaccatggaggtggaagagttcttgaaagaagctgcagtcatgaaagagatcaaacaccctaacct

GUSB --------------------------------------------------------------------------------

BCR --------------------------------------------------------------------------------

BCR-ABL1 1313 accttgaaggaggacaccatggaggtggaagagttcttgaaagaagctgcagtcatgaaagagatcaaacaccctaacct

Lab1 5921 ggtgcggtcgactctagatgcatgctcgagcggccgccagtgtgatggat

Lab2 5921 ggtgcggtcgactctagatgcatgctcgagcggccgccagtgtgatggat

GUSB --------------------------------------------------

BCR --------------------------------------------------

BCR-ABL1 1393 ggtgc---------------------------------------------

**Suppl. Fig 2:** Restriction analysis of pIRMM-0099. Lane 1: pIRMM-0099 restricted with *Hinc*II (expected fragments: 4573 bp, 860 bp and 544 bp); Lane 2: pIRMM-0099 restricted with *Pvu*II (expected fragments: 2372 bp, 1965 bp, 709 bp, 598 bp, 195 bp and 132 bp; Lane 3: pIRMM-0099 restricted with *Xho*I (plasmid linearisation expected); Lane 4: 1 kb DNA ladder (Invitrogen, Life Technologies Europe, Gent, BE).


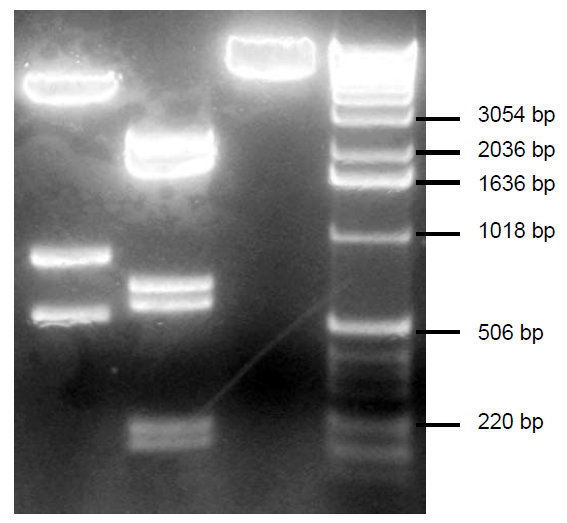


**Suppl. Figure 3.** Homogeneity study results for the copy number concentration of ERM-AD623a-f as determined by qPCR. The bars indicate the standard deviation from 3 replicates.

**Suppl. Figure 4.** Short term stability study results for the copy number concentration of ERM-AD623a-f as determined by qPCR.

.

**Suppl. Figure 5.** Long term stability study results for the copy number concentration of ERM-AD623a-f as determined by qPCR.

**Suppl. Figure 6.**  Stability study results of the copy number concentration of ERM-AD623a,c,f after several freeze/thaw cycles as determined by qPCR.

**Suppl. Figure 7.** The mean absolute copy number concentration of *BCR-ABL1* measured for two common cDNA samples using ERM-AD623 as a calibrator in the small scale suitability study.


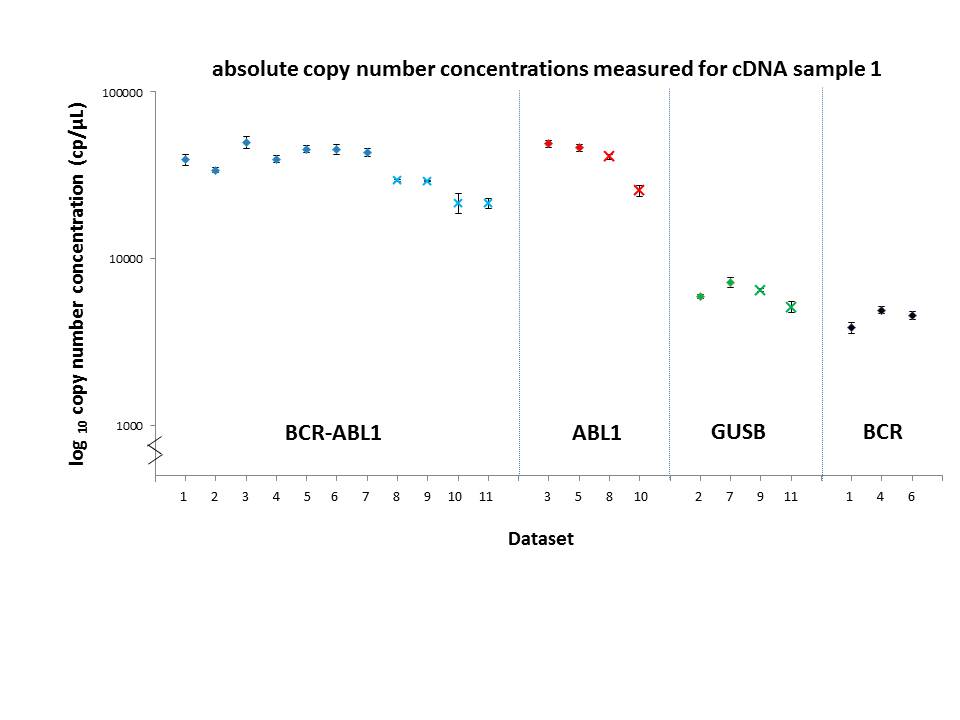


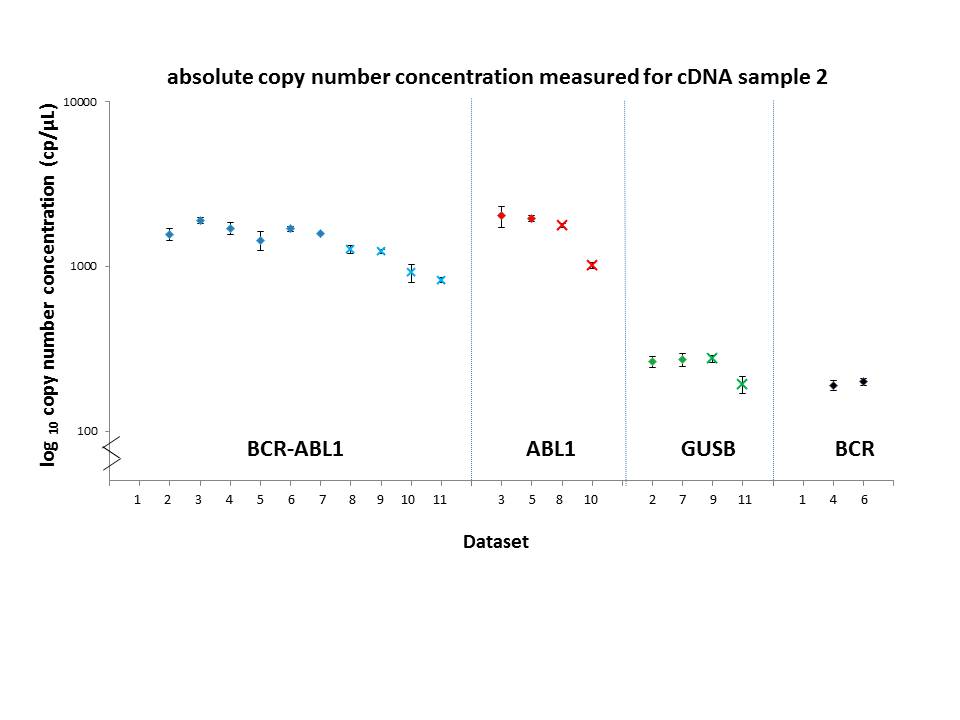


The error bars represent the standard deviation. The squares indicated the results obtained with methods based on the Taqman technology (datasets 1 to 7) and the crosses indicated the results obtained with LightCycler-based methods (datasets 8 to 11).

**Suppl. Figure 8**. The mean copy number ratios *BCR-ABL1*/CG measured for two common cDNA samples using ERM-AD623 as a calibrator in the small scale suitability study.


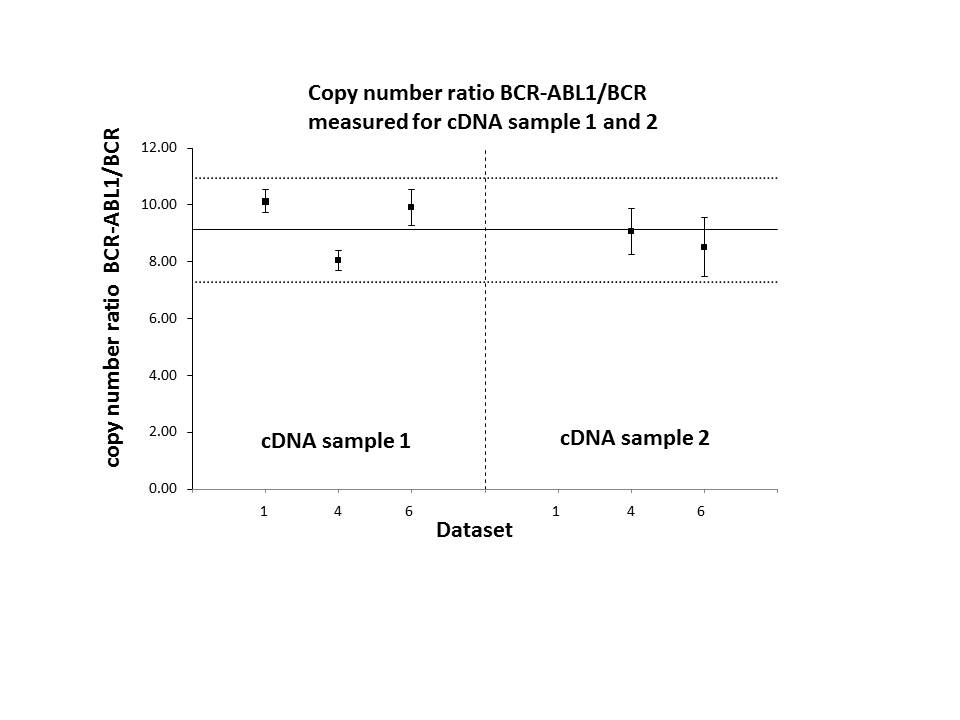


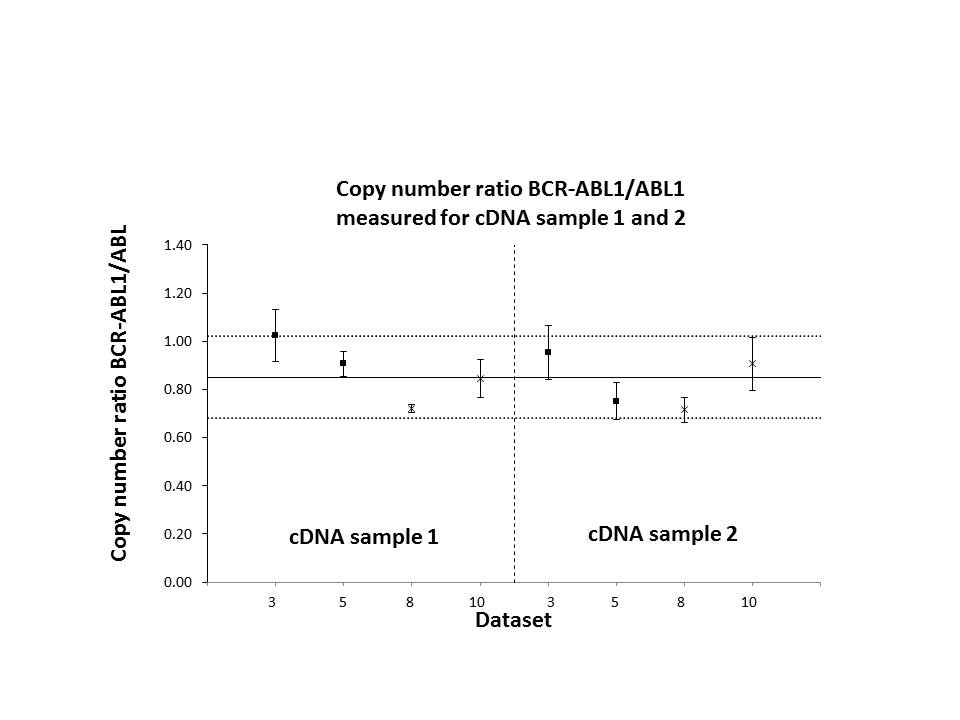


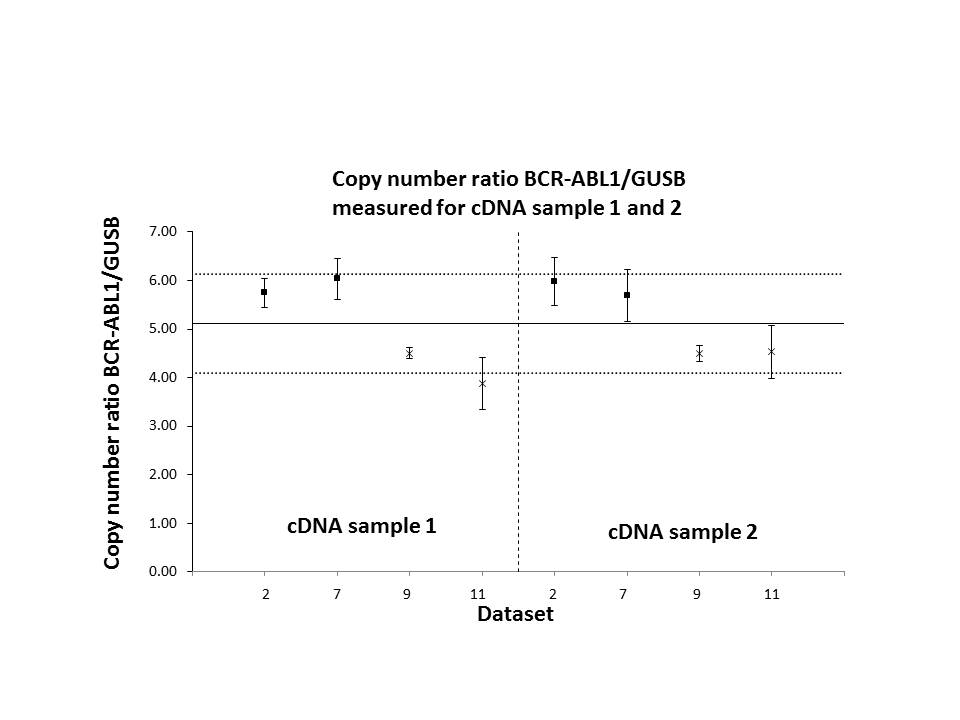


The error bars represent the standard deviation. The black squares indicated the results obtained with methods based on the Taqman technology (datasets 1 to 7) and the crosses indicated the results obtained with LightCycler-based methods (datasets 8 to 11). The full lines mark the average *BCR-ABL1*/CG copy number ratio and the dashed line mark 80% and 120% of this average value (the 1.2-fold range).

**SUPPLEMENTARY TABLES:**

**Suppl. Table 1**. Sequences of primers used to amplify cloned fragments of *BCR-ABL1* e14a2, *BCR* and *GUSB* and the sequences of the primers and probes used to amplify the *BCR-ABL1* and the *ABL1* target in duplex in the digital PCR and qPCR measurements.

| **PCR primers used for cloned fragments** | | | | |
| --- | --- | --- | --- | --- |
| **PCR target** | **Primers** | **Sequence** | | **Amplicon size** |
| *BCR-ABL1* e14a2 | Forward primer  Reverse primer | CGAGAGTCGACTCTCTGCACCAAGCTCAAGA  CGAGAGTCGACctgcaccaggttagggtgtt | | **1397bp** |
| *BCR* | Forward primer  Reverse primer | GTCCACTCAGCCACTGGATT  CAAGGACCAGCTGTCAGTCA | | **813bp** |
| *GUSB* | Forward primer  Reverse primer | TTTCCGTACCAGCCACTACC  GTAAACGGGCTGTTTTCCAA | | **963bp** |
| **Primers and Probes used in duplex for the digital PCR and the qPCR measurements** | | | |  |
| **PCR target** | **Primer/ probe** | **Sequence** | **Concentration in PCR [nM]** | **Amplicon size** |
| *BCR-ABL1* | Forward primer | 5'-TCCGCTGACCATCAAYAAGGA-3' | 300 | 149 |
|  | Reverse primer | 5'-CACTCAGACCCTGAGGCTCAA-3' | 300 |  |
|  | Probe | 5'-(6-VIC)-CCCTTCAGCGGCCAGTAG CATCTGA-(MGB)-3' | 200 |  |
| *ABL1* | Forward primer | 5'-TGGAGATAACACTCTAAGCATAAC TAAAGGT-3' | 300 | 122 |
|  | Reverse primer | 5'-GATGTAGTTGCTTGGGACCCA-3' | 300 |  |
|  | Probe | 5'-(6-FAM)-CCATTTTTGGTTTGGGCTT CACACCATT-(TAMRA)-3' | 200 |  |

**Suppl. Table 2.** Duplex digital PCR measurements for *BCR-ABL1/GUSB* and *BCR-ABL1/BCR* show that each insert is present only once in pIRMM0099.

| CRM | Copy number ratio *BCR-ABL/GUSB* ± *s* | Copy number ratio *BCR-ABL/BCR* ± *s* |
| --- | --- | --- |
| ERM-AD623a | 1.015 ± 0.017 | 1.000 ± 0.013 |
| ERM-AD623b | 0.994 ± 0.016 | 1.019 ± 0.015 |
| ERM-AD623c | 0.990 ± 0.012 | 1.012 ± 0.033 |
| ERM-AD623d | 1.008 ± 0.022 | 0.994 ± 0.021 |
| ERM-AD623e | 1.000 ± 0.018 | 1.005 ± 0.020 |
| ERM-AD623f | 0.989 ± 0.074 | 0.966 ± 0.050 |

These ratios were the mean ratios obtained from five panels of one digital PCR array ± the standard deviation s.

**Suppl. Table 3.** The DNA mass concentrations and the estimated copy number concentrations obtained for the stock solution of linearised plasmid pIRMM0099 using three different methods.

| Method | Number of replicates | Mean c_DNA_ ± *s* [ng/µL] | Copy number concentration ± *s* [10^10^ cp/µL] |
| --- | --- | --- | --- |
| Fluorometry (picogreen) | 6 | 210.5 ± 2.8 (measured) | 3.43 ± 0.05 |
| Spectrophotometry | 3 | 214.0 ± 5.6 (measured) | 3.49 ± 0.09 |
| dPCR | 2 | 205.1 ± 17.8 (estimated) | 3.34 ± 0.29 |

**Suppl. Table 4. Characterisation study for the copy number concentration of ERM-AD623a-f**

ERM-AD623a

| Laboratory number | Measurement number | Result | *s*_within labs_ [%] | *s*_between labs_ [%] |  |
| --- | --- | --- | --- | --- | --- |
|  | 1 | 973463 |  |  |  |
|  | 2 | 884230 |  |  |  |
| 1 | 3 | 1091851 |  |  |  |
|  | 4 | 1216974 | 12.66 |  |  |
|  | 5 | 1085891 |  |  |  |
|  | 6 | 1244606 |  |  |  |
|  | 7 | 990555 |  |  |  |
|  | 8 | 1076161 | 10.11 |  |  |
| 2 | 9 | 1075208 |  | n.c. |  |
|  | 10 | n.a. |  |  |  |
|  | 11 | 1318816 |  |  |  |
|  | 12 | 1203224 |  |  |  |
|  | 13 | 850527 |  |  |  |
|  | 14 | 971317 |  |  |  |
| 3 | 15 | 888295 | 16.88 |  |  |
|  | 16 | 1020358 |  |  |  |
|  | 17 | 1302875 |  |  |  |
|  | 18 | 1187144 |  |  |  |
| all measurements | | 1081264 | 13.03 |  |  |

*MS*_between labs_: mean of squares between the results from different laboratories obtained from the ANOVA test. MS_within labs_:  mean of squares within the results from one laboratory obtained from the ANOVA test, S_within labs_ standard deviation within the results from one laboratory obtained from the ANOVA test *S*_between labs_: standard deviation between the results from different laboratories obtained from the ANOVA test. n.a.: not accepted, n.c.: cannot be calculated as *MS*_between_ < *MS*_within_

ERM-AD623b

| Laboratory number | Measurement  number | Result | *s*_within labs_ [%] | *s*_between labs_ [%] |  |
| --- | --- | --- | --- | --- | --- |
|  | 1 | 89381 |  |  |  |
|  | 2 | 83629 |  |  |  |
| 1 | 3 | 101269 |  |  |  |
|  | 4 | 120777 | 17.86 |  |  |
|  | 5 | 120027 |  |  |  |
|  | 6 | 131755 |  |  |  |
|  | 7 | 100105 |  |  |  |
|  | 8 | 108482 | 11.64 |  |  |
| 2 | 9 | 107063 |  | 5.03 |  |
|  | 10 | 131156 |  |  |  |
|  | 11 | 130595 |  |  |  |
|  | 12 | 118884 |  |  |  |
|  | 13 | 84084 |  |  |  |
|  | 14 | 93263 |  |  |  |
| 3 | 15 | 87540 | 14.17 |  |  |
|  | 16 | 99239 |  |  |  |
|  | 17 | 120806 |  |  |  |
|  | 18 | 110646 |  |  |  |
| all measurements | | 107706 | 15.12 |  |  |

ERM-AD623c

| Laboratory number | Measurement  number | Result | *s*_within labs_ [%] | *s*_between labs_ [%] |  |
| --- | --- | --- | --- | --- | --- |
|  | 1 | 8619.9 |  |  |  |
|  | 2 | 10290.2 |  |  |  |
| 1 | 3 | 11565.9 |  |  |  |
|  | 4 | 10984.8 | 9.46 |  |  |
|  | 5 | 10522.5 |  |  |  |
|  | 6 | 10582.7 |  |  |  |
|  | 7 | 9067.9 |  |  |  |
|  | 8 | 10612.3 | 12.95 |  |  |
| 2 | 9 | 10842.0 |  | 6.25 |  |
|  | 10 | 13205.1 |  |  |  |
|  | 11 | 12194.6 |  |  |  |
|  | 12 | 10508.6 |  |  |  |
|  | 13 | 9232.2 |  |  |  |
|  | 14 | 8475.1 |  |  |  |
| 3 | 15 | 9956.1 | 11.73 |  |  |
|  | 16 | 8255.1 |  |  |  |
|  | 17 | 9512.8 |  |  |  |
|  | 18 | 11344.6 |  |  |  |
| all measurements | | 10320.7 | 12.68 |  |  |

ERM-AD623d

| Laboratory number | Measurement  number | Result | *s*_within labs_ [%] | *s*_between labs_ [%] |  |
| --- | --- | --- | --- | --- | --- |
|  | 1 | 939.7 |  |  |  |
|  | 2 | 1033.0 |  |  |  |
| 1 | 3 | 1113.7 |  |  |  |
|  | 4 | 1074.2 | 6.40 |  |  |
|  | 5 | 1100.1 |  |  |  |
|  | 6 | 984.1 |  |  |  |
|  | 7 | 869.1 |  |  |  |
|  | 8 | 1119.0 | 11.61 |  |  |
| 2 | 9 | 1077.3 |  | 6.70 |  |
|  | 10 | 1244.1 |  |  |  |
|  | 11 | 1156.7 |  |  |  |
|  | 12 | 1039.1 |  |  |  |
|  | 13 | 893.2 |  |  |  |
|  | 14 | 838.2 |  |  |  |
| 3 | 15 | 1028.9 | 10.15 |  |  |
|  | 16 | 818.2 |  |  |  |
|  | 17 | 960.8 |  |  |  |
|  | 18 | 1041.7 |  |  |  |
| all measurements | | 1018.4 | 11.20 |  |  |

ERM-AD623e

| Laboratory number | Measurement  number | Result | *s*_within labs_ [%] | *s*_between labs_ [%] |  |
| --- | --- | --- | --- | --- | --- |
|  | 1 | 90.1 |  |  |  |
|  | 2 | 92.2 |  |  |  |
| 1 | 3 | 104.6 |  |  |  |
|  | 4 | 120.4 | 11.40 |  |  |
|  | 5 | 114.1 |  |  |  |
|  | 6 | 99.9 |  |  |  |
|  | 7 | 94.3 |  |  |  |
|  | 8 | 110.0 | 11.43 |  |  |
| 2 | 9 | 104.3 |  | 8.22 |  |
|  | 10 | 115.7 |  |  |  |
|  | 11 | 127.5 |  |  |  |
|  | 12 | 127.7 |  |  |  |
|  | 13 | 92.8 |  |  |  |
|  | 14 | 82.7 |  |  |  |
| 3 | 15 | 88.3 | 8.72 |  |  |
|  | 16 | 95.5 |  |  |  |
|  | 17 | 95.8 |  |  |  |
|  | 18 | 107.9 |  |  |  |
| all measurements | | 103.5 | 12.81 |  |  |

ERM-AD623f

| Laboratory number | Measurement  number | Result | *s*_within labs_ [%] | *s*_between labs_ [%] |  |
| --- | --- | --- | --- | --- | --- |
|  | 1 | n.a. |  |  |  |
|  | 2 | 8.72 |  |  |  |
| 1 | 3 | 9.60 | 15.43 |  |  |
|  | 4 | 12.63 |  |  |  |
|  | 5 | 9.66 |  |  |  |
|  | 6 | 8.61 |  |  |  |
|  | 7 | 8 |  |  |  |
|  | 8 | 11.75 |  |  |  |
| 2 | 9 | 9.6 | 18.72 | 5.78 |  |
|  | 10 | 9.43 |  |  |  |
|  | 11 | 13.35 |  |  |  |
|  | 12 | 13.3 |  |  |  |
|  | 13 | 9.16 |  |  |  |
|  | 14 | 8.88 |  |  |  |
| 3 | 15 | 7.78 | 6.68 |  |  |
|  | 16 | 9.66 |  |  |  |
|  | 17 | 10.54 |  |  |  |
|  | 18 | 8.72 |  |  |  |
| all measurements | | 9.96 | 15.96 |  |  |

n.a.: not accepted

**Suppl Table 5.** Standard deviations of measurements within and between labs and the standard deviation of all measurements.

| CRM | Laboratory number | *s*_within lab, rel_ [%] | *s*_between labs, rel_ [%] | *s*_all measurements, rel_ [%] |
| --- | --- | --- | --- | --- |
| ERM-AD623a | 1 | 12.66 | n.c. | 13.03 |
|  | 2 | 10.11 |  |  |
|  | 3 | 16.88 |  |  |
| ERM-AD623b | 1 | 17.86 | 5.03 | 15.12 |
|  | 2 | 11.64 |  |  |
|  | 3 | 14.17 |  |  |
| ERM-AD623c | 1 | 9.46 | 6.25 | 12.68 |
|  | 2 | 12.95 |  |  |
|  | 3 | 11.73 |  |  |
| ERM-AD623d | 1 | 6.40 | 6.70 | 11.20 |
|  | 2 | 11.61 |  |  |
|  | 3 | 10.15 |  |  |
| ERM-AD623e | 1 | 11.40 | 8.22 | 12.81 |
|  | 2 | 11.43 |  |  |
|  | 3 | 8.72 |  |  |
| ERM-AD623f | 1 | 15.43 | 5.78 | 15.96 |
|  | 2 | 18.72 |  |  |
|  | 3 | 6.68 |  |  |

**Suppl. Table 6:** Methods used for the small scale commutability study

| **Laboratory number** | **Control gene** | **Reference of qPCR method** | **qPCR instrument** |
| --- | --- | --- | --- |
| **1** | *BCR* | [1] | ABI 7000 (Applied Biosystems) |
| **2** | *ABL1* | [2,3] | ABI 7000 (Applied Biosystems) |
| **3** | *GUSB* | [2,3] | ABI 7500 FAST (Applied Biosystems) |
| **4** | *ABL1* | [2,3] | ABI 7900 HT (Applied Biosystems) |
| **5** | *ABL1* | [2,3] | ABI 7900 HT (Applied Biosystems) |
|  | *GUSB* | [2,3] |  |
| **6** | *ABL1* | Home made | LightCycler480 (Roche) |
|  | *GUSB* | Home made |  |
| **7** | *ABL1* | [4] | LightCycler1.5 (Roche) |
|  | *GUSB* | [5] |  |
| **8** | *BCR* | [1] | ABI7500 (Applied Biosystems) |
| **9** | *ABL1* | [2,3] | Rotorgene 6000 (Corbett/QIAGEN) |
|  | *BCR* | [1] |  |
|  | *GUSB* | [2,3] |  |

**Suppl. Table 7:** Hardware, source of plasmid and control gene analysed for the 57 laboratories participating in the second commutability study.

|  | | **Number of labs** |
| --- | --- | --- |
| **RNA Extraction** | | |
|  | Trizol | 31 |
|  | RLT | 25 |
|  | Trizol and RLT | 1 |
| **Real Time PCR Machine** | | |
|  | ABI Prism 7000 SDS | 3 |
|  | ABI 7300 | 1 |
|  | ABI 7500 | 10 |
|  | ABI 7500 fast | 5 |
|  | ABI 7900 | 6 |
|  | ABI 7900 HT | 6 |
|  | Lightcycler 1.5 | 5 |
|  | Lightcycler 2.0 | 2 |
|  | Lightcycler 480 | 8 |
|  | Corbett RG3000 | 3 |
|  | Corbett RG6000 | 4 |
|  | Mx3000 Stratagene | 1 |
|  | Viia7 Dx | 1 |
|  | Step one plus | 1 |
|  | Not known | 1 |
| **Plasmid source** | | |
|  | Ipsogen | 36 |
|  | pME-2 (Mannheim) | 7 |
|  | Wessex | 4 |
|  | Nanogen | 1 |
|  | In house | 5 |
|  | Not applicable | 4 |
| **Control gene** | | |
|  | *ABL1* | 50 |
|  | *GUSB* | 5 |
|  | *ABL1* and *GUSB* | 1 |
|  | Other | 1 |

**SUPPLEMENTARY REFERENCES:**

1) S. Branford, T.P. Hughes, Z. Rudzki, Monitoring chronic myeloid leukaemia therapy by real-time quantitative PCR in blood is a reliable alternative to bone marrow cytogenetics. Br J Haematol. 107 (1999) 587-99

2) J. Gabert, E. Beillard, V.H. van der Velden, W. Bi, D. Grimwade, N. Pallisgaard, G. Barbany, G. Cazzaniga, J.M. Cayuela, H. Cavé, F. Pane, J.L. Aerts, D. De Micheli, X. Thirion, V. Pradel, M. González, S. Viehmann, M. Malec, G. Saglio, J.J. van Dongen, Standardization and quality control studies of 'real-time' quantitative reverse transcriptase polymerase chain reaction of fusion gene transcripts for residual disease detection in leukemia - a Europe Against Cancer program. Leukemia. 17 (2003) 2318-57.

3) E. Beillard, N. Pallisgaard, V.H. van der Velden, W. Bi, R. Dee, E. van der Schoot, E. Delabesse, E. Macintyre, E. Gottardi, G. Saglio, F. Watzinger, T. Lion, J.J. van Dongen, P. Hokland, J. Gabert, Evaluation of candidate control genes for diagnosis and residual disease detection in leukemic patients using 'real-time' quantitative reverse-transcriptase polymerase chain reaction (RQ-PCR) - a Europe against cancer program, Leukemia. 17 (2003) 2474-86.

4) M Emig, S Saussele, H Wittor, A Weisser, A Reiter, A Willer, U Berger,R Hehlmann, NC Cross, A Hochhaus. Accurate and rapid analysis of residual disease in patients with CML using specific fluorescent hybridization probes for real time quantitative RT-PCR. Leukemia. 13 (1999) 1825-32.

5) M.C. Müller, P. Erben, G. Saglio, E. Gottardi, C.G. Nyvold, T. Schenk, T. Ernst, S. Lauber, J. Kruth, R. Hehlmann, A. Hochhaus; European LeukemiaNet. Harmonization of BCR-ABL mRNA quantification using a uniform multifunctional control plasmid in 37 international laboratories. Leukemia 22 (2008) 96-102.
